# Daam-, FMNL-, and mDia-family formins jointly regulate cortical actin dynamics in melanoma cells

**DOI:** 10.1186/s12964-026-03229-9

**Published:** 2026-10-01

**Authors:** Thomas Kaufmann, Jonas Scholz, Agnes Csiszár, Marius Karger, Moritz Walkenbach, Klemens Rottner, Rudolf Merkel, Ute Curth, Jan Faix

**Affiliations:** 1 Institute for Biophysical Chemistry, Hannover Medical School, Carl-Neuberg-Strasse 1, Hannover, 30625 Germany; 2 Institute of Biological Information Processing 2: Mechanobiology, Forschungszentrum Jülich GmbH, Wilhelm-Johnen-Straße, Jülich, 52428 Germany; 3 Molecular Cell Biology Group, Helmholtz Centre for Infection Research, Inhoffenstrasse 7, Brunswick, 38124 Germany; 4 Division of Molecular Cell Biology, Institute for Cell and Neurobiology, Technische Universität Braunschweig, Spielmannstraße 7, Braunschweig, 38106 Germany; 5 Braunschweig Integrated Centre of Systems Biology (BRICS), Rebenring 56, Braunschweig, 38106 Germany

## Abstract

The actin-rich cell cortex is a viscoelastic structure essential for processes such as cell migration and division, yet the full spectrum of actin assembly factors that build and regulate the cortex in mammalian cells remains incompletely defined. While the Arp2/3 complex generates actin networks mostly comprising short, branched filaments, formins like mDia1 and mDia3 assemble long, linear filaments capable of withstanding the high mechanical load produced by myosin-driven contraction at the cell rear. To obtain a complete inventory of formins that regulate mechanics of the contractile cortex in B16-F1 melanoma cells, we systematically screened Diaphanous-related formins (DRFs) for interactions with Rho-family GTPases regulating contractility. This screen led to the identification of Daam1/2 and FMNL2/3 as cortical formins, since active variants of these formins as well as the endogenous proteins prominently localized to the rear cortex of polarized B16-F1 cells. Yeast two-hybrid (Y2H) and GST pull-down assays revealed prominent interactions of Daam1/2 with active RhoA, RhoB, RhoC, RhoD, and Cdc42, and weaker binding to Rac1 and Rif. Pyrene-actin polymerization and single-filament TIRF assays further demonstrated that these GTPases effectively relieve autoinhibition of Daam-family formins, with Daam1 and 2 emerging as a potent elongators. In line with this, CRISPR/Cas9-generated Daam1/2 double-knockout cells exhibited multiple defects, including formation of multiple leading edges, impaired random 2D migration, reduced focal adhesion (FA) turnover and inefficient rear retraction. However, contrary to earlier work, we found no evidence that Daam1/2 regulate the formation of finger-like protrusions. By comparison, FMNL2 appears to act as a dual-role formin at both the cell front and rear, interacting with and being activated by several GTPases linked to contractility and protrusion. Analytical ultracentrifugation further clarified previous inconsistencies by showing that the affinity of FMNL2 for GTPases, particularly for Rac1, depends on the length of its N-terminal fragment and the presence of the full FH3 domain. Finally, we show that cells lacking Daam1/2 and mDia1/3 display pronounced cortical defects, including reduced rigidity, excessive blebbing, and delayed cytokinesis, demonstrating that these formins cooperate to preserve cortical architecture and function.

**Supplementary Information:**

The online version contains supplementary material available at https://doi.org/10.1186/s12964-026-03229-9.

## Introduction

Eukaryotic cells are enclosed by a thin, actin-rich cortex located beneath the plasma membrane to modulate cell shape, define their mechanical properties and provide resilience against external forces [8, 19, 87]. The three-dimensional architecture of this tightly crosslinked viscoelastic network is sculpted by a wide range of actin-binding and regulatory proteins, including nonmuscle myosin 2 (NM2), which generates forces that produce cortical tension and, through tension gradients, drives cell shape changes such as those occurring during migration or cytokinesis [20, 52]. Efficient force generation further relies on the structural organization, density, and length of cortical actin filaments, which are orchestrated by dedicated actin assembly factors and accessory proteins [21].

Cellular actin polymerization is primarily driven by two classes of nucleators: the Arp2/3 complex and formins. The Arp2/3 complex, best known for nucleating dense, branched actin networks in lamellipodia at the leading edge, acts downstream of the WAVE regulatory complex (WRC) and Rac subfamily GTPase signaling [44, 72]. In contrast, formins are dimeric multidomain proteins that nucleate and elongate linear actin filaments [22], such as those found in actin bundles, the cleavage furrow or the cell cortex [4, 35, 80]. All mammalian formins harbor a proline-rich formin homology 1 (FH1) domain to recruit profilin (PFN)-actin complexes for subsequent filament elongation by the adjacent FH2 domain [62]. A subfamily of the 15 known formins referred to as Diaphanous-related formins (DRFs) are tightly regulated [27, 78]. Due to regulatory elements within their N- and C-terminal regions, these proteins are intrinsically autoinhibited [81]. Binding of Rho-family GTPases to the N-terminal GTPase-binding domain (GBD) disrupts the interaction between the adjacent formin homology 3 (FH3) domain and the C-terminal diaphanous autoregulatory domain (DAD), thereby relieving autoinhibition to activate the formin, enabling engagement with downstream effectors [46, 69]. Post-translational modifications, such as phosphorylation in the C-terminal region of mDia2 (Diaph3), have also been reported to weaken the interaction between the GBD-FH3 and the DAD [40, 84]. Interestingly, previous studies suggested that in blebbing M2 melanoma and mitotic HeLa cells, most cortical actin filaments arise from the combined activities of the Arp2/3 complex and the DRF mDia1 (Diaph1), though contributions from the DRFs FH1/FH2 domain-containing protein 1 (FHOD1) and Dishevelled-associated activator of morphogenesis 1 (Daam1) could not be excluded [12]. Nevertheless, this and other studies indicate that the Arp2/3 complex plays a comparably minor role in cortical cell mechanics, especially relative to formins such as mDia1 [28]. Moreover, unlike mitotic or detached cells, polarized cells typically exhibit a spatial segregation of their propulsive and contractile actin machineries. At the cell front, the Arp2/3 complex generates highly branched networks of short actin filaments with a pronounced directional bias toward the plasma membrane and along the axis of cell movement. This predominantly non-contractile network exhibits greater effective stiffness in the direction of protrusion, thereby supporting forward membrane advancement. In contrast, cortical formins contribute to the assembly of a relatively continuous and dense contractile network of long, unbranched actin filaments at the cell rear. Here, filaments are arranged with substantially less directional bias and span multiple orientations, supporting actomyosin-dependent contractility and mechanical integrity of the cortex [20, 64, 87]. Consistently, active variants of mDia1 and mDia3 (Diaph2) were shown to localize prominently to the contractile rear cortex of highly polarized B16-F1 mouse melanoma cells, and CRISPR/Cas9-mediated elimination of these DRFs triggered the formation of multiple protrusive fronts along with substantial defects in cell polarization and 2D migration [48]. In highly adherent NIH 3T3 fibroblasts, combined loss of mDia1/3 caused a hyperstretched morphology with diminished, multi-tipped lamellipodia, enlarged FAs, and prominent high-strain stress fibers (SFs) that frequently ruptured to form tension- and contractility-induced macroapertures (TCMs), relieving membrane tension and boosting lamellipodial protrusion [76]. In Sertoli cells, their loss likewise markedly impaired architecture of the cortical F-actin meshwork, but instead led to the formation of non-contractile F-actin bundles [73], together indicating that respective, resulting phenotypes are cell-type dependent, at least in part. The situation with the third and final mDia-subfamily member mDia2 (Diaph3), is more complex. Studies using ectopic expression of constitutively-active mDia2-EGFP fusion constructs under control of the CMV promotor reported accumulation at the leading edge, with a stark bias for filopodia tips and implicated mDia2 in filopodia formation [10, 91], whereas Watanabe and colleagues detected mDia2 at the cell cortex, spindle, and midbody during mitosis in NIH 3T3 cells [89]. Following mitosis, however, mDia2 is largely degraded through an ubiquitin-dependent pathway [24]. Consistent with this, endogenous mDia2 was shown to be largely absent from defined actin-based structures in interphase cells, including B16-F1 and NIH 3T3 cells [34]. Thus, mDia2 appears unlikely to account for the striking difference in phenotype caused by combined loss of mDia1 and mDia3, which is substantially less severe in B16-F1 cells than in NIH 3T3 fibroblasts [48, 76]. This discrepancy instead suggested that additional cortical formins operate in B16-F1 cells to safeguard the mechanical properties of the contractile rear cortex. Given that the molecular mechanisms governing cortex mechanics remain incompletely understood, identifying the full complement of cortical formins in mammalian cells and dissecting their respective contributions therefore remains an important objective.

## Results

### Defining the repertoire of cortical formins

**Fig. 1:**
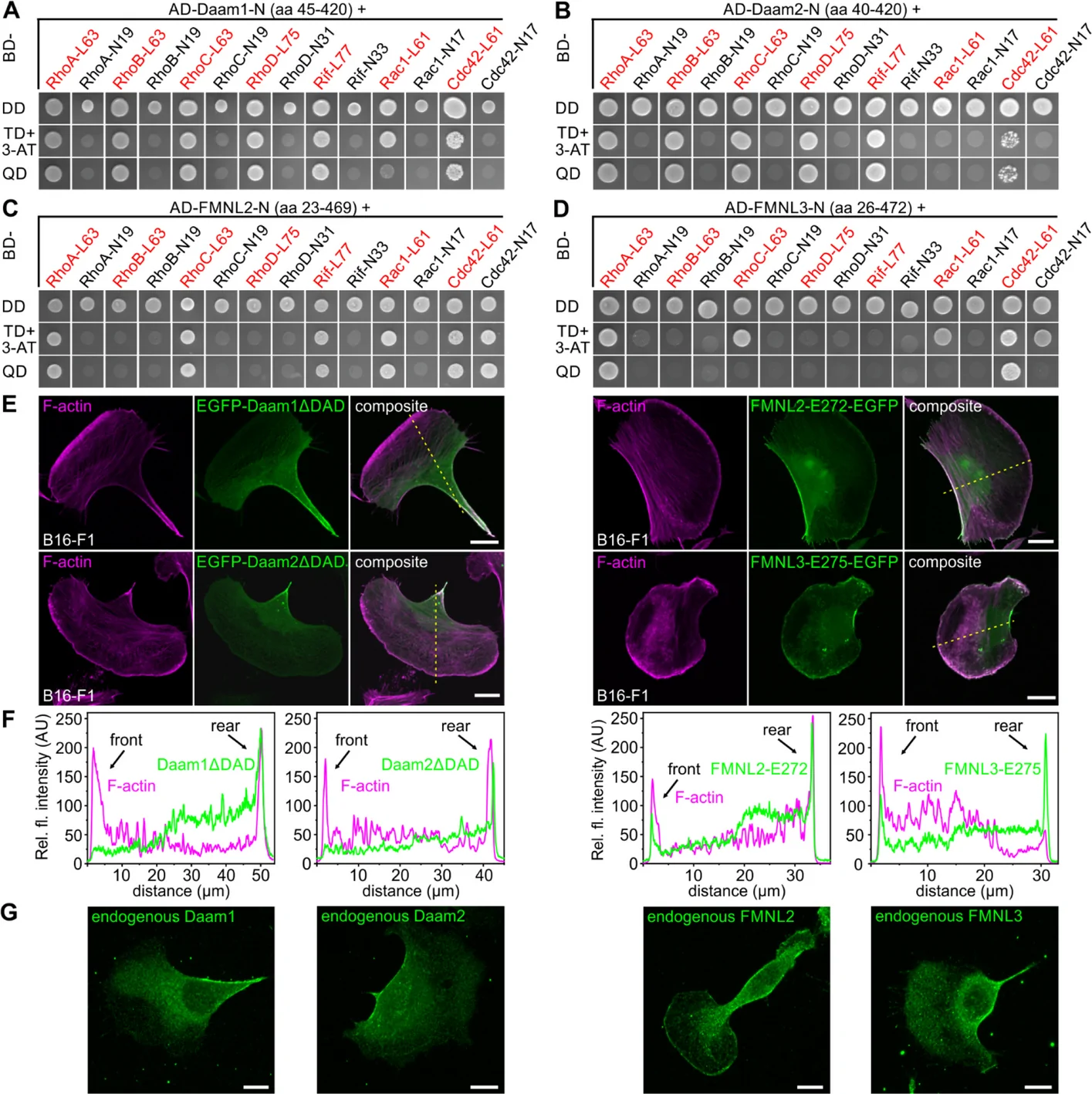
Daam and FMNL formins bind active Rho-family GTPases and localize to the rear cortex. **A-D** Yeast two-hybrid screens confirmed interactions of respective GBD/FH3 domains, as indicated, with constitutively-active Rho-family GTPases (red). Dominant-negative GTPases (black) served as controls. Yeast cells were transformed with constructs as indicated and selected for the presence of prey and bait plasmids by growth on double dropout (DD) media lacking leucine and tryptophan. Protein–protein interactions were monitored by growth on selective triple-dropout (TD) media additionally lacking histidine in the presence of 3 mM 3-amino-1,2,4-triazole (3-AT) and on quadruple-dropout (QD) media additionally lacking histidine and adenine. AD, GAL4-activation domain; BD, GAL4-DNA-binding domain; aa, amino-acid residues. **E** Subcellular localization of constitutively-active Daam1, Daam2, FMNL2, and FMNL3 variants fused to EGFP in polarized B16-F1 cells migrating on laminin. The F-actin cytoskeleton was visualized with phalloidin. Note prominent accumulation of Daam1/2 at the rear cortex, and of FMNL2/3 in both lamellipodial protrusions and rear cortex. Scale bars, 10 µm. Dashed lines indicate positions used to derive intensity profiles of the respective proteins across cells. **F** Intensity profiles depicting respective formin (green) and F-actin (magenta) intensity in cells shown in (**E**). Arrows indicate the positions of the front and rear of the cells. **G** Representative images of B16-F1 cells migrating on laminin, fixed with TCA and stained for endogenous Daam1, Daam2, FMNL2 and FMNL3. Scale bars, 10 µm. E and G, brightest point projection of confocal stacks

To assess the complete repertoire of cortical formins, we systematically screened putative candidates of the remaining DRFs encompassing Daam-, FMNL- and FHOD-family formins with Rho-GTPases implicated in contractility using Y2H assays. To this end, we employed N-terminal GBD-FH3 fragments of Daam1 and 2, FMNL2 and 3 as well as FHOD1 as prey. FMNL1 and FHOD3 were excluded from analysis because previous studies found them to be primarily expressed in the hematopoietic system and cardiac muscle, respectively [4, 9]. The DRF mDia2 was omitted due to its lack of cortical association in interphase cells [34]. The small GTPases potentially associated with contractility, RhoA, RhoB, RhoC, RhoD and Rif (also known as RhoF), were used as bait, while the more distantly related GTPases Cdc42 and Rac1, best known for driving the formation of cell protrusions, served as controls. Strong and specific interactions with active GTPases were identified for four of the five tested formins. Under the most stringent growth conditions on selective media, Daam1 interacted with the constitutively-active variants of RhoA, RhoB, RhoC, RhoD, Rif and Cdc42, while it failed to interact with the dominant-negative variants of these GTPases (Fig. 1A), confirming specificity of interactions. Similar results were obtained for Daam2 (Fig. 1B). FMNL2 exhibited interactions with active RhoA, RhoC, Rif, Rac1 and Cdc42, albeit it also showed a comparable interaction with dominant-negative Cdc42 (Fig. 1C). FMNL3 had the lowest number of binding partners and interacted only with active RhoA and Cdc42 (Fig. 1D). Presumably, due to its inability to fold properly in yeast, no interactions were found with FHOD1 (Supplementary Fig. 1).

Next, we examined the subcellular localization of constitutively-active formin variants, either lacking the DAD region or containing point mutations that relieve autoinhibition, by analyzing cells expressing low levels of EGFP-tagged constructs in B16-F1 mouse melanoma cells, which exhibit a highly polarized morphology with a distinct leading edge and trailing end. The fixed cells were additionally stained with phalloidin to visualize the F-actin cytoskeleton. Consistent with previous findings [29], constitutively-active FHOD1 did not localize to the cell cortex but instead promoted massive SF formation and accumulated within those structures (Supplementary Fig. 1). In contrast, similar to active mDia1 and 3 [48], we found that ectopically expressed, constitutively-active Daam1/2ΔDAD accumulated markedly at the contractile cell cortex in the rear, while active FMNL2-E272/FMNL3-E275 localized to both the front and the rear (Fig. 1E). This distribution of Daam- and FMNL-family formins in B16-F1 cells was further supported by corresponding EGFP- and phalloidin-intensity profiles measured along lines spanning the cells (Fig. 1F). Notably, Daam1 accumulation at the rear cortex is partly consistent with previous reports of localization to contractile SFs, the centrosome and cortical regions at the cell membrane [3, 12, 51], while another study reported enrichment along the shafts and at the tips of filopodia and microspikes at the leading edge [38]. Furthermore, active FMNL2/3 are hitherto known for their functions in filopodia and at the lamellipodium tip, where they contribute to force generation during protrusion of the cell front [9, 39]. Notwithstanding this, the rear cortical localization of the Daam- and FMNL formins was confirmed by staining the respective endogenous proteins in fixed cells using our specific antibodies (Fig. 1G and Supplementary Fig. 2). The prominent cortical localization was additionally confirmed in NIH 3T3 fibroblasts, despite their less pronounced polarization along the front-rear axis compared with B16-F1 cells (Supplementary Fig. 3), suggesting a conserved cellular function in distinct cell types. Interestingly, we also observed a moderate accumulation of Daam2 in the nucleus of B16-F1 cells and NIH 3T3 fibroblasts (Fig. 1 and Supplementary Fig. 3), potentially pointing to an additional function in the nucleus as suggested recently [42]. Importantly, RNA-Seq analysis further revealed that mDia1, Daam1/2, and FMNL2/3 formins are expressed at comparably high levels in B16-F1 cells, whereas mDia3 was expressed at lower levels (Supplementary Fig. 4). Collectively, these data strongly support a cooperative role for these formins in the regulation of cell cortex dynamics in B16-F1 cells.

### Biochemical characterization of Daam1 and 2

**Fig. 2:**
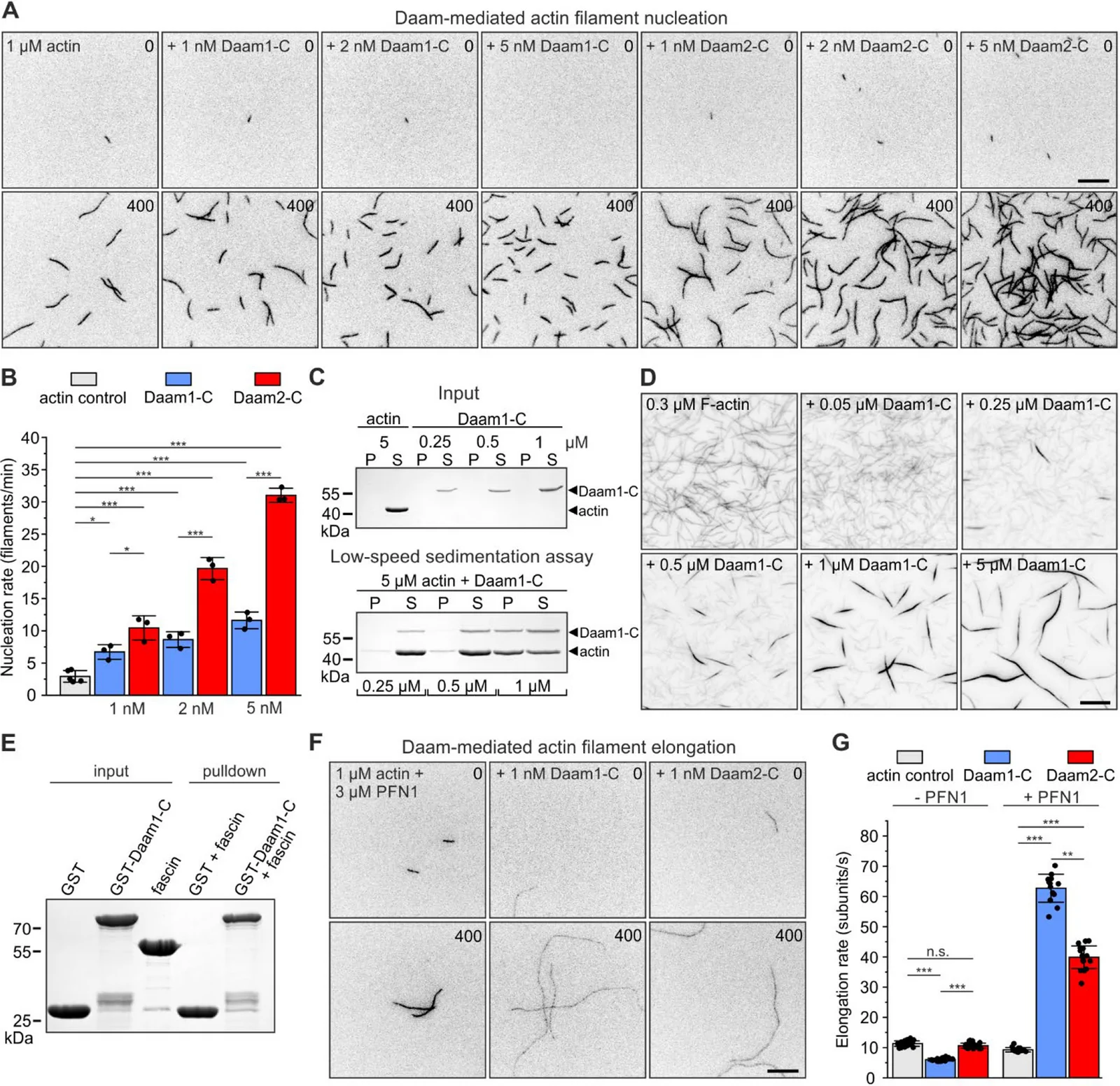
Daam family formins are potent actin assembly factors. **A** Still images from time-lapse TIRF movies using 1 µM actin (20% Atto488-labeled) in the presence of Daam1-C or Daam2-C at the concentrations as indicated, at 0 s and 400 s. Scale bar, 10 µm. **B** Quantification of the actin nucleation rates in the absence and presence of the indicated Daam1/2-C concentrations. The number of filaments was plotted over time and subjected to linear regression, with the nucleation rates calculated from the slope of the fitted lines. *P*-values were determined using one-way ANOVA. Data correspond to means ± SD. At least three independent movies per condition were analyzed. **C** Input (top) and low-speed sedimentation assays (bottom) of actin and Daam1-C at concentrations as indicated. Proteins in the pellet (P) and supernatant (S) fractions recovered after centrifugation at 18,000 × g were stained with Coomassie blue. **D** Daam1-C exhibits moderate actin filament bundling in TIRF assays using 0.3 µM pre-polymerized F-actin (20% Atto488-labeled) in the presence of increasing concentrations of Daam1-C. Images represent stills from time-lapse TIRF movies after 300 s. Scale bar, 10 µm. **E** Daam1-C does not interact with fascin. Input of purified proteins and GST pull-down experiment using GST, GST-Daam1-C and tag-free fascin, as indicated. **F** Still images from time-lapse TIRF movies using 1 µM actin (20% Atto488-labeled) in the presence of 3 µM PFN1 and 1 nM Daam1-C or Daam2-C, respectively. Scale bar, 10 µm. **G** Quantification of actin elongation rates of Daam1-C or Daam2-C in the absence and presence of PFN1. At least 13 filaments were analyzed from at least three independent movies. *P*-values were determined using Kruskal–Wallis ANOVA followed by Dunn’s multiple comparison test. Data are shown as mean ± SD. * *P* < 0.05, ** P < 0.01, *** *P* < 0.001, ns: not significant

As opposed to mDia- and FMNL-family formins, which have been extensively studied [9, 39, 43, 47], the biochemical properties of the Daam formins have remained only partially characterized [36, 38, 61], with currently no available data on Daam2. To compare how Daam1 and Daam2 affect the nucleation and elongation of actin filaments, we generated the corresponding cDNAs from B16-F1 mouse melanoma cells, and then expressed and purified the C-terminal halves of Daam1 (residues 560–1068, Daam1-C) and Daam2 (526–1067, Daam2-C) encompassing the FH1 and the FH2 domain along with all remaining C-terminal residues (Supplementary Fig. 5). The FH1 domains of both Daam constructs, mediating recruitment of profilin-actin for subsequent filament assembly, harbored two of the three stretches of consecutive proline residues to facilitate bacterial expression. To examine formin-mediated actin assembly at the single filament level in real time, we then performed total internal reflection fluorescence (TIRF) microscopy to visualize and quantify the birth and growth of filaments in the absence or presence of PFN1, which is ubiquitously expressed at high levels in most cell-types [82]. In the absence of PFN1, both formin fragments stimulated actin nucleation in a concentration-dependent manner and produced significantly more filaments compared to actin control, with Daam2 exhibiting an approximately threefold higher activity than Daam1 (Fig. 2A, B,Supplementary Fig. 6, Movies 1 and 2). Nevertheless, even at higher Daam1-C concentrations (250 nM), we could not observe filament bundling in the typical actin polymerization TIRF assay, unlike previous reports using C-terminal fragments of human Daam1 [38, 61], presumably due to the potent nucleation activity of the murine formin (Movie 3). Thus, we explored its bundling activity using a low-speed sedimentation assay. As shown in Fig. 2C, murine Daam1-C promoted moderate, yet clearly detectable filament bundling at relatively high concentrations. This activity was further confirmed at the single filament level in TIRF assays using preformed actin filaments to avoid nucleation (Fig. 2D). To our surprise, however, we did not observe any bundling activity by Daam2-C (Supplementary Fig. 7). Moreover, although Jaiswal et al. reported a direct interaction between Daam1 and the actin-bundling protein fascin, with additional studies using FLIM-FRET or co-immunoprecipitation further supporting an association between the two proteins [33, 57], we did not detect binding between purified GST-Daam1-C and fascin in our pulldown experiments (Fig. 2E). Next, we assessed how Daam1 and 2 affect filament elongation. In control reactions containing 1 µM G-actin spiked with 20% Atto488-labeled monomers, spontaneously formed filaments elongated at a rate of 11.3 ± 0.9 subunits/s. The addition of 3 µM PFN1 slightly reduced this rate to 9.3 ± 0.7 subunits/s. However, when C-terminal fragments of Daam1 or Daam2 were included along with PFN1, we observed the formation of dim and rapidly growing filaments, consistent with previous findings that formins preferentially incorporate unlabeled actin under these conditions [43]. Quantifications revealed that these filaments elongated at significantly higher rates of 62.7 ± 4.7 subunits/s for Daam1-C and 39.8 ± 3.7 subunits/s for Daam2-C compared to actin alone control (Fig. 2F, G and Movie 4), Thus, despite their similar activities, Daam1 is a more potent filament elongator, while Daam2 is more effective in the nucleation of actin filaments.

### Activation of Daam1 and FMNL2 by Rho-family GTPases* in vitro*

**Fig. 3:**
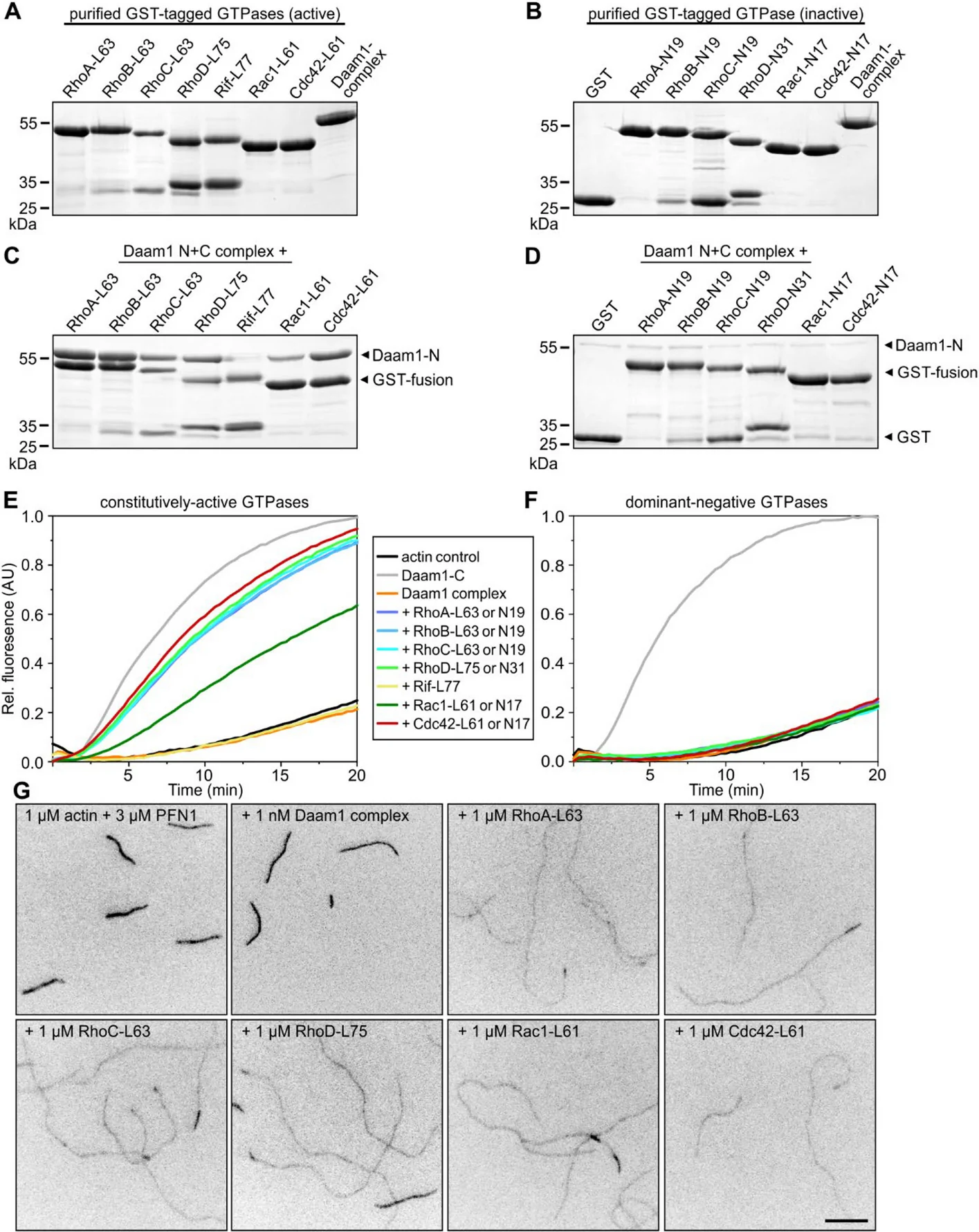
Active Rho-family GTPases release autoinhibition of the inactive Daam1 complex. **A**, **B** Purified Daam1 complex (Daam1-N + Daam1-C) and GST-GTPase variants after SDS-PAGE and Coomassie-staining. **C**, **D** GST pull-down experiment using constitutively-active or inactive GST-GTPases to confirm direct and largely GTP binding-dependent interactions with Daam1-N. **E**, **F** Rho-GTPases interacting with Daam1-N are able to release autoinhibition of the Daam1 complex and promote actin filament assembly as assessed by pyrene actin polymerization assays. Reactions contained 2 µM actin (5% pyrene-labeled), 25 nM Daam1-C or 25 nM Daam1 complex with 1 µM GST-GTPases, as indicated. Curves represent mean values from three independent measurements. **G** Representative TIRF-microscopy images after 400 s from time-lapse movies confirming Daam1 complex activation by active Rho-GTPases at the single filament level. Actin polymerization of 1 µM actin (20% Atto488-labeled) with proteins at concentrations as indicated was imaged for 10 min at 5 s intervals. Scale bar, 10 µm

Next, we investigated the GTPase-specific interactions of Daam-family formins using GST pull-down experiments and actin pyrene assays with representative GTPases identified in our Y2H screen. As Daam1 and 2 showed virtually identical interactions, subsequent analyses were carried out with Daam1 as a representative. Because addition of the N-terminal region of Daam1 (Daam1-N) to its C-terminal fragment did not yield a fully inactive formin, we co-expressed both fragments in *E. coli* to aid formation of an autoinhibited Daam1 sandwich complex (Fig. 3A, B). Since the two fragments were nearly identical in size, we first confirmed presence of both polypeptides in the purified complex by immunoblotting with specific antibodies (Supplementary Fig. 8). Then, we expressed and purified recombinant GST-fusion proteins of the respective Rho-family GTPases in their constitutively-active or dominant-negative forms (Fig. 3A, B). Consistent with the Y2H data, the active forms of RhoA-D, Cdc42 and Rac1 interacted with Daam1 in the GST pull-down experiment, whereas Rif did not (Fig. 3C). This lack of interaction is likely attributable to improper folding of Rif in E. coli, which is consistent with data showing that Rif cannot be expressed as functional protein in bacteria [86], although previous studies had reported this approach to be successful [30, 36, 37]. Nevertheless, we observed a distinct, though moderate accumulation of active Rif-L77 fused to EGFP in the rear cortex of B16-F1 cells (Supplementary Fig. 9). By contrast, none of the analyzed dominant-negative GTPase variants bound Daam1, confirming the specificity of the observed interactions (Fig. 3D). Next, we explored the ability of the GTPases to relieve autoinhibition of the complex by pyrene actin assays. Unlike 25 nM Daam1-C, which effectively promoted the polymerization of 2 µM G-actin, 25 nM of the Daam1 complex mediated polymerization kinetics indistinguishable from actin control, demonstrating autoinhibition of the Daam1 sandwich complex (Fig. 3E). Conversely, constitutively-active RhoA-D, Cdc42, and Rac1 were effective to relieve autoinhibition of the formin complex, with Rac1 showing the weakest activation. By comparison, none of the GTPases was able to activate the complex in their dominant-negative form (Fig. 3F). Finally, we employed TIRF imaging to visualize the release of complex autoinhibition on the single filament level in real time. In control reactions with 1 µM actin (20% Atto488-labeled), 3 µM PFN1 and the presence or absence of 1 nM Daam1 complex, a comparable number of bright filaments was found, all elongating at about 9 subunits per second, confirming autoinhibition of the Daam1 complex (Fig. 3G). Upon addition of 1 µM active RhoA-D, Rac1 or Cdc42 to the inactive Daam1 complex, autoinhibition was effectively released, as evidenced by the formation of dim, rapidly growing actin filaments elongating with a rate of about 60 subunits/sec, closely resembling the velocity observed with the unregulated Daam1-C fragment alone (Fig. 3G, Supplementary Fig. 10 and Movie 5). Of note, reconstitution of the autoinhibited Daam2 sandwich complex was precluded because the Daam2 N-terminal fragment could not be purified as soluble protein from bacterial extracts.

Given the controversial views on whether FMNL2 activity in cells is primarily regulated by RhoC, Rac1 or Cdc42 [9, 31, 41, 45]. we additionally generated and purified an N-terminal FMNL2 fragment (FMNL2-N1, residues 1–526), along with an inactive formin complex composed of FMNL2-N1 and FMNL2-C (residues 558–1092). We then examined interactions and regulatory mechanisms of FMNL2 involving the Rho-family GTPases identified in our Y2H screen, along with RhoB and RhoD, again employing GST pull-down assays and TIRF imaging. Notably, in the pull-down experiments, FMNL2-N1 bound strongly to active RhoC and Cdc42, as well as to RhoA and Rac1, but showed no detectable interaction with active RhoD (Supplementary Fig. 11 A). Unexpectedly, we also observed a moderate interaction with active RhoB, which had not been detected in the Y2H assay. However, the interaction with dominant-negative Cdc42 in the pull-down assays aligned with our findings in the Y2H screen (Supplementary Fig. 11B). To validate the most prominent interactions, we then tested whether active RhoA, RhoC, Rac1, and Cdc42 could relieve autoinhibition of the inactive FMNL2 complex by TIRF microscopy. Similar to the Daam1 complex, 1 nM of the FMNL2 complex reconstituted from N- and C-terminal fragments was inactive, with filament growth and numbers indistinguishable from the control in the presence of 1 µM actin and 3 µM PFN1 (Supplementary Fig. 11 C, Movie 6). Upon addition of 1 µM active GTPases to the FMNL2 complex, autoinhibition was relieved in all cases, with Cdc42 followed by Rac1 being most effective, as indicated by the large fraction of fast-growing, dim filaments while RhoA and RhoC appeared to have a weaker impact (Supplementary Fig. 11 C, Movie 6). Notably, despite interaction of FMNL2 with dominant-negative Cdc42 in Y2H and pull-down assay, this GTPase variant failed to release autoinhibition of the inactive FMNL2 complex. Combined, these data thus not only confirm activation of FMNL2 by active Cdc42, Rac1 and RhoC, at least *in vitro*, but also expand the repertoire of regulatory GTPases to RhoA and potentially Rif, although the latter could not be tested in our GST pull-down and TIRF assays.

### Analysis of FMNL2-GTPase interactions by analytic ultracentrifugation

The identification of multiple FMNL2-regulatory GTPases prompted us to examine the interaction of FMNL2-N1 with active RhoA, Rac1 and Cdc42 using analytical ultracentrifugation (AUC) to better evaluate the functional relevance of these GTPases. We have chosen to examine RhoA over RhoC because it is more efficiently expressed in *E. coli* and shares identical switch I and II regions, which determine the interaction with downstream effectors [2]. While active RhoA, Rac1 and Cdc42 sedimented as single, globular, monomeric species with an *s*-value of about 2.1 S, FMNL2-N1 displayed a broad distribution of *s*-values ranging from about 7 to 15 S (Supplementary Fig. 12A), suggesting the formation of various oligomerization states, most likely ranging from dimers to octamers, similar to previous observations for mDia1 and FHOD1 [54, 77]. The addition of a twofold molar excess of RhoA, Rac1 or Cdc42 resulted in clear shifts in the FMNL2-N1 *s*-value distribution towards higher *s*-values in all three cases (Supplementary Fig. 12B-D). Although this clearly demonstrates a direct interaction between these GTPases and FMNL2-N1, the heterogeneity of FMNL2-N1 made it impossible to quantify these interactions. Thus, to determine the relative affinities of FMNL2 for the three GTPases, we used a shorter N-terminal construct, referred to here as FMNL2-N2 (residues 1–379), which lacks part of the FH3 domain and has previously been co-crystallized with active Cdc42 as a monomer [45]. Consistently, FMNL2-N2 sedimented as a single species with 3 S (Supplementary Fig. 12A) and *c*(*s*) analysis yielded a molar mass of 42 kg/mol, indicating that it also forms monomers in solution. The addition of active RhoA, Rac1 or Cdc42 resulted in clear shifts of the FMNL2-N2 peak with increasing concentrations of the GTPases (Supplementary Fig. 12E-G). However, the affinities of the 1:1 complexes differed markedly: full saturation was achieved with just a twofold molar excess of Cdc42, while even a fivefold excess of Rac1 failed to reach saturation. This trend was confirmed by quantitative analysis, yielding *K*_D_ values of ≤ 0.5 µM for Cdc42, 14 µM for RhoA, and ≥ 150 µM for Rac1 (Supplementary Fig. 12H). The marked difference between Cdc42 and Rac1 confirmed here could be narrowed down to three residues within the interaction surface of the GTPase with the used formin construct [45]. Notably however, although both formin constructs included the complete GBD, active RhoA and Rac1 bound significantly better to the longer FMNL2 variant harboring the entire FH3 domain.

### Loss of Daam-family formins impairs random 2D-migration but does not affect microspike formation

**Fig. 4:**
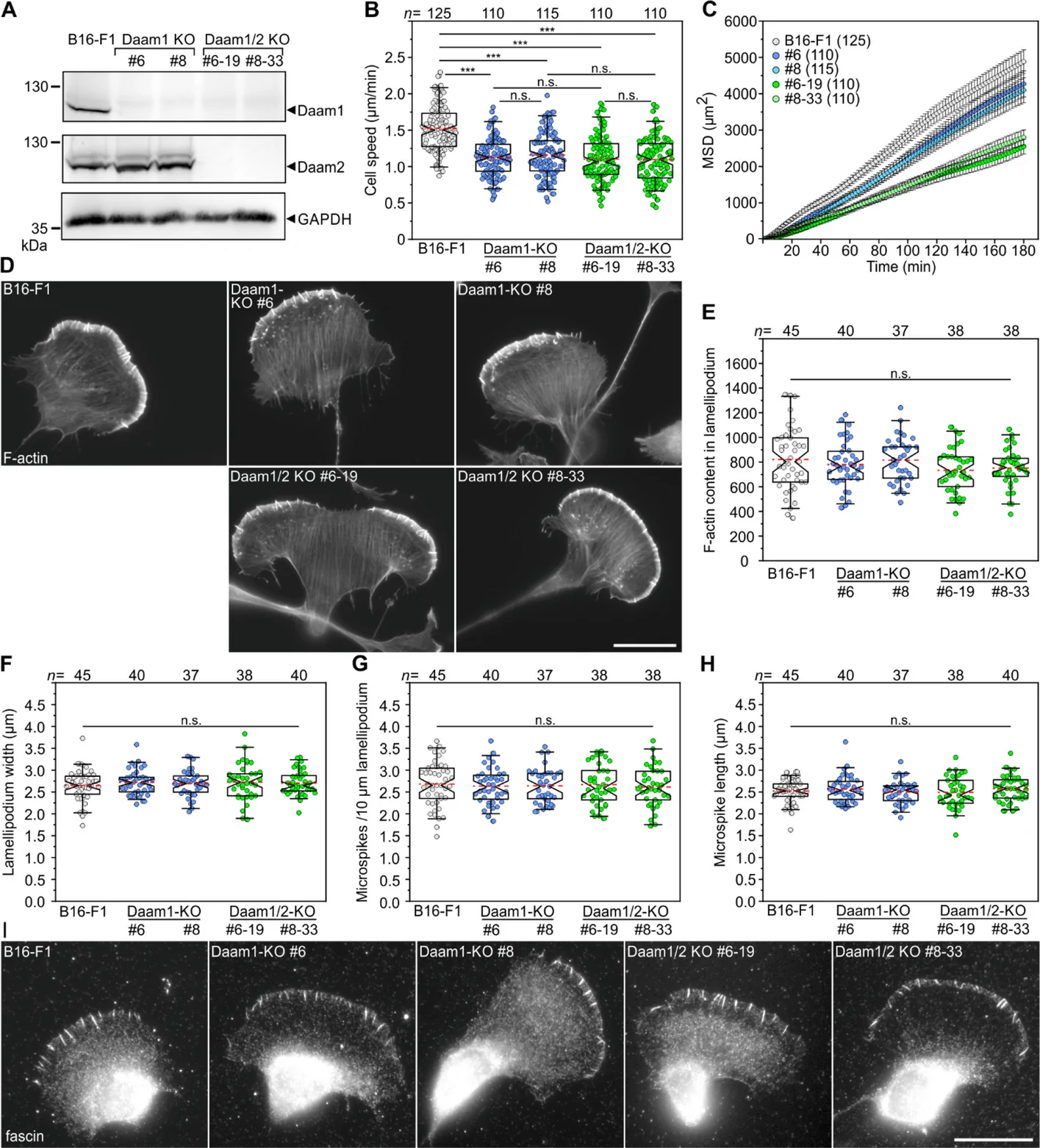
Loss of Daam-family formins impairs cell migration, but not microspike formation in B16-F1 cells. **A** Loss of Daam1 and Daam2 by CRISPR-Cas9 in B16-F1 cells was confirmed by immunoblotting using specific antibodies. GAPDH was used as loading control. **B** Elimination of Daam1 resulted in a defect in 2D cell migration on laminin, whereas the additional loss of Daam2 produced little further effect. *P*-values were determined using one-way ANOVA followed by Tukey’s multiple comparison test. **C** Analyses of mean square displacement of B16-F1 control *versus* mutant cells. Data represent means ± SEM. **D** Representative images of B16-F1 control and mutant cells stained with phalloidin for the F-actin cytoskeleton. Scale bar, 20 µm. **E** Quantifications of lamellipodial F-actin content and **F** lamellipodium width in control and mutant cells. **G** Quantifications of microspike numbers and **H** length in control and mutant cells. *P*-values were determined using one-way ANOVA followed by Tukey’s multiple comparison test. **I** Representative images of B16-F1, Daam1-KO and Daam1/2-KO cells stained for the microspike marker protein fascin. Scale bar, 20 µm. Boxes in box plots indicate 50% (25 to 75%) and whiskers 90% (5 to 95%) of all measurements, with dashed red lines depicting arithmetic means. Medians are highlighted by indentation of boxes. *n*, number of cells analyzed. *** *P* < 0.001, ns: not significant

To examine the cellular functions of Daam-family formins, we eliminated Daam1 and Daam2 expression by CRISPR/Cas9 technology consecutively in B16-F1 mouse melanoma cells. Respective protein loss in independent clonal cell lines was confirmed by immunoblotting and TIDE analysis (Fig. 4A and Supplementary Fig. 13). Random 2D-migration rates of B16-F1 wild-type (control) and mutant cells on laminin were then analyzed by phase-contrast, time-lapse microscopy. Consecutive Daam member removal decreased 2D-migration rate noticeably in the single Daam-KO mutant compared to control, while additional loss of Daam2 had only a minor effect (Fig. 4B). Then, we calculated the mean square displacement (MSD) to assess their effective directional movement. All Daam mutants showed progressively reduced MSD levels compared to control, with the double mutants exhibiting the most pronounced impairment (Fig. 4C). As 2D migration on flat surfaces primarily depends on actin assembly in the lamellipodium at the cell front and cell substrate adhesion, we first examined actin filament (F-actin) distribution in B16-F1 cells and representative Daam mutants after phalloidin staining. Surprisingly, however, both single and double Daam mutants exhibited canonical lamellipodia with numerous microspikes, similar to those formed by B16-F1 control cells (Fig. 4D), challenging previous conclusions about Daam1 functions in protrusive actin bundle formation at the leading edge [38]. Consistently, neither lamellipodial F-actin content nor lamellipodial width differed detectably between mutant and control cells (Fig. 4E, F), and quantification of microspike density and length revealed no significant changes across genotypes (Fig. 4G, H). This was further supported by the nearly identical fascin staining of microspikes in wild-type, single-mutant, and double-mutant cells (Fig. 4I), as well as by live-cell imaging of B16-F1 and Daam1/2-KO cells expressing EGFP-fascin (Movie 7).

### Loss of Daam1/2 impairs protrusion persistence despite unchanged lamellipodial markers

**Fig. 5:**
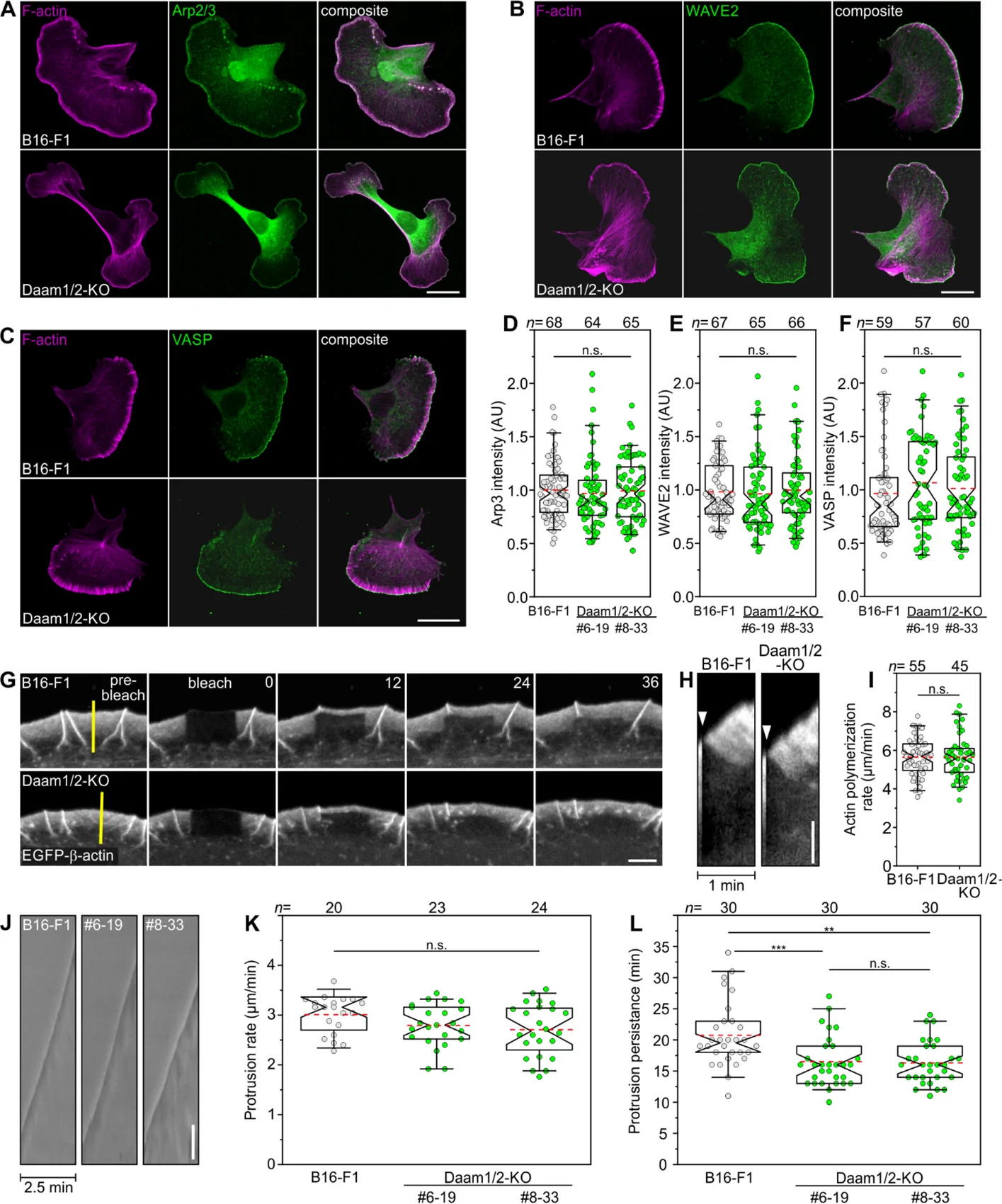
Loss of Daam-family formins does not affect lamellipodial parameters except protrusion persistence. **A-C** Representative images of B16-F1 and Daam1/2-KO cells seeded on laminin and stained for the F-actin cytoskeleton together with lamellipodial markers. (**A**) Arp2/3 complex, (**B**) WAVE2 and (**C**) VASP. Scale bar, 20 µm. **D-F** Quantification of relative Arp3, WAVE and VASP intensities in the lamellipodium of control and mutant cells. *P*-values were determined using Kruskal–Wallis ANOVA followed by Dunn’s multiple comparison test. **G** FRAP of EGFP-ß-actin in lamellipodia of B16-F1 and Daam1/2-KO cells. Numbers in post-bleach images correspond to s. Scale bar, 5 µm. **H** Kymographs derived from control and Daam1/2-KO movies as indicated by the yellow line in (**G**) are shown. Arrowheads indicate time points of bleaching. Scale bar, 2 µm **I** Quantification of the actin polymerization rate in wild-type and mutant cells expressing EGFP-ß-actin. *P*-values were determined by two-tailed unpaired Student’s t-test. **J** Kymographs of representative phase-contrast movies of B16-F1 and Daam1/2-KO cells. Scale bar, 2.5 µm. **K**, **L** Quantification of protrusion rates and protrusion persistence in control and mutant cells. *P*-values for protrusion were determined using one-way ANOVA, while *P*-values for persistence were calculated using the Kruskal–Wallis ANOVA test followed by Dunn’s multiple comparison test. Boxes in box plots indicate 50% (25 to 75%) and whiskers 90% (5 to 95%) of all measurements, with dashed red lines depicting the means. Medians are highlighted by indentation of boxes. *n*, number of cells analyzed. ** P < 0.01, *** *P* < 0.001, ns: not significant. A-C, brightest point projection of confocal stacks

To further examine whether loss of Daam1/2 function affects accumulation of key components of the actin assembly machinery at the leading edge, we immunolabelled cells for the Arp2/3 complex, the WRC component WAVE2, and the actin polymerase VASP (Fig. 5A-C). However, we found that all three displayed canonical localizations mostly throughout lamellipodia (Arp2/3) and at lamellipodial tips (WAVE2 and VASP), with virtually identical intensity and distribution in both wild-type and the double-mutant cells (Fig. 5D-F). To exclude potential compensation of lamellipodial defects by other formins, we then compared B16-F1 control and Daam1/2 double-knockout cells by RNA-Seq analysis. However, we did not detect any noticeable compensatory upregulation of other formins in the double mutant compared with control B16-F1 cells (Supplementary Fig. S14).

To test if dynamics of actin assembly in lamellipodia is altered, we next assessed lamellipodial actin network assembly rates in control and Daam1/2-KO cells, ectopically expressing EGFP-β-actin, by fluorescence recovery after photobleaching (FRAP) analysis. Notably, lamellipodial actin network translocation and actin polymerization rates in the double mutant were virtually unchanged as compared to control (Fig. 5G-I, Movie 8). Since lamellipodial actin assembly does not necessarily translate into equally effective protrusion, as e.g. in FMNL2/3-KOs [39], we asked whether or to what extent lamellipodial protrusion was affected upon Daam1/2 removal. To address this, we imaged wild-type and Daam1/2-KO cells migrating randomly on laminin by phase-contrast microscopy and determined respective protrusion rates by kymograph analyses (Fig. 5J). However, quantifications revealed that lamellipodial protrusion was also unchanged in Daam1/2-KO cells as compared to controls (Fig. 5K), suggesting together with the FRAP and immunolabeling data that the reduced migration of the double mutants cannot be explained by changes in lamellipodial actin dynamics. Nevertheless, despite these highly comparable lamellipodial parameters, the persistence of individual lamellipodia was reduced by about 20% in both Daam1/2-KO mutants relative to controls (Fig. 5L), indicating that the decrease in 2D migration upon Daam1/2 loss may result from other, more indirect effects on protrusion duration, such as altered cell shape, adhesion, or both.

### Daam-deficient cells exhibit multiple fronts and defects in focal adhesion dynamics

**Fig. 6:**
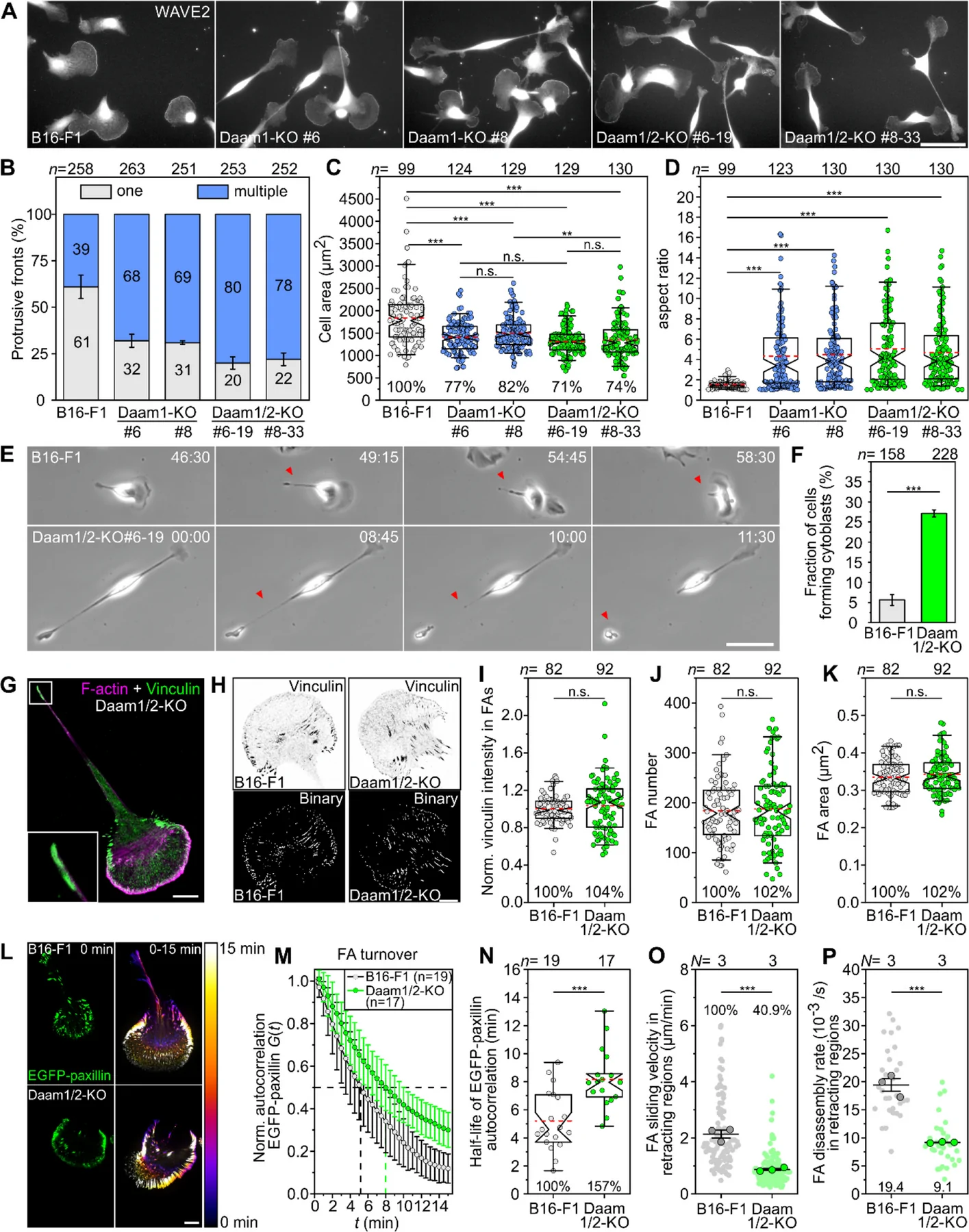
Combined loss of Daam1/2 alters cell morphology and perturbs adhesion. **A** Representative images of B16-F1 control and mutant cells stained for WAVE2 to visualize protrusive fronts. Scale bar, 50 µm. **B** Percentage of control and mutant cells displaying one or multiple protrusive fronts. **C** Quantification of the cell area. **D** Quantification of aspect ratio. *P*-values (C, D) were determined using Kruskal–Wallis ANOVA followed by Dunn’s multiple comparison test. **E** Still images from time-lapse movies of control and Daam1/2-KO cells migrating on laminin at time points (min:s), as indicated. Red arrowheads highlight respective retraction of the cell body. Scale bar, 50 µm. **F** Fraction of wild-type and mutant cells forming cytoplasts, as quantified from at least three independent movies each. Boxes and whiskers represent mean ± SD. *P*-value was determined using the two-tailed unpaired Student’s *t*-test. **G** Representative image of an elongated Daam1/2-KO mutant cell stained for the FA marker protein vinculin and the F-actin cytoskeleton. Inset, enlarged image of boxed region. **H** Representative images of control and mutant cells stained for vinculin before (top) and after binarization (bottom). Scale bar (F, G), 10 µm. **I** Quantification of normalized vinculin intensities in FAs. **J** Quantification of FA number. **K** Quantification of FA area. *P*-values were determined using the Mann–Whitney-*U*-test for (I, J) and the two-tailed unpaired Student’s *t*-test for (K). **L** Representative images from time-lapse, TIRF movies of control and Daam1/2-KO cells migrating on laminin and displaying EGFP-paxillin accumulation in FAs (left); data correspond to Movie S10. Temporal, color-coded time stacks of the same cells over a 15 min period (right). Scale bar, 10 μm. Bar on far right depicts color-coded time stamp. **M** Quantification of FA turnover by normalized autocorrelation of EGFP-paxillin fluorescence over time. Data are shown as mean ± SD. **N** Quantification of half-life of EGFP-paxillin autocorrelation. *P*-values were determined by two-tailed unpaired Student’s *t*-test. **O** Quantification of FA sliding velocity in retracting regions. **P** Quantification of the FA disassembly rate in retracting regions. Small dots in (O, P) represent measurements from individual FAs, while large dots represent the arithmetic mean of each independent biological replicate. Lines and error bars indicate the mean ± SEM. A total of 102 FAs from seven individual cells per cell line were analyzed in (O), and a total of 30 FAs from four B16-F1 cells and five Daam1/2-KO cells were analyzed in (P). *N*, number of independent biological replicates. *P*-values were determined by two-tailed unpaired Student’s *t*-test based on the arithmetic means of the biological independent replicates. Boxes in box plots indicate 50% (25 to 75%) and whiskers 90% (5 to 95%) of all measurements, with dashed red lines depicting the means. Medians are highlighted by indentation of boxes. *n*, number of cells analyzed. ** P < 0.01, *** *P* < 0.001, ns: not significant

Loss of the cortical formins ForA, ForE, and ForH in *Dictyostelium* amoebae, as well as of mDia1 and mDia3 in B16-F1 cells and NIH 3T3 fibroblasts, has previously been shown to result in the formation of multiple leading edges [48, 76]. Of note, in mDia1/3-deficient B16-F1 cells, this multiple-front phenotype was associated with impaired random 2D migration on laminin and polarization [48]. To assess the impact of Daam-family formins on cell morphology, we fixed B16-F1 control and Daam-mutant cells randomly migrating on laminin, stained them for the lamellipodial marker WAVE2 and recorded images at low magnification (Fig. 6A). Analysis of the stainings revealed changes in cell shape and, notably, a higher frequency of cells with multiple fronts in Daam1 single mutants. This phenotype was further exacerbated in Daam1/2 double-knockout cells, which frequently formed up to five lamellipodia pointing in different directions simultaneously, whereas B16-F1 control cells typically displayed only one or two protrusive fronts (Fig. 6A, B). Interestingly, consecutive removal of the Daam-family formins also correlated with a reduction in cell area to 77% and 82% in the single knockout mutants, and to 71% and 74% in the double mutants, respectively, compared to control cells (Fig. 6C). Moreover, the elongated cell shape of the Daam mutants resulted in a markedly increased aspect ratio, demonstrating a pronounced defect in cell polarization (Fig. 6D). To gain more insights into the migratory behavior and dynamic cell shape changes of the double mutant, we then imaged Daam1/2-deficient cells at higher magnification by time-lapse, phase-contrast microscopy and compared their properties to that of B16-F1 control cells. Unlike migrating control cells, which typically retracted their trailing edge into the cell body to drive effective cell migration, Daam1/2 double-knockout cells exhibited defects in rear-end retraction, frequently forming unusually long cellular tethers that eventually ruptured, detached from the cell body, and gave rise to cytoplasts (Fig. 6E-F, Movie 9). Thus, we reasoned that the double-mutant might either have altered adhesive properties, a weakened cell cortex that fails to generate enough force to disassemble old FAs at the rear, or both. Consistent with this notion, immunolabeling of Daam1/2-KO cells forming long tethers revealed a prominent accumulation of the FA marker vinculin in the cellular fragment connected by the tether to the cell body, indicating impaired dissolution of mature FAs (Fig. 6G). Thus, to systematically assess the role of Daam-family formins in FA formation, control and Daam1/2-KO cells migrating on laminin were immunolabeled for vinculin, and images captured under identical settings analyzed using a web-based FA analysis tool [7], enabling a global and unbiased evaluation of multiple FA parameters (Fig. 6H). However, in variance to mDia1/3-deficient NIH 3T3 fibroblasts forming exaggerated FAs [76], vinculin intensity, FA number, and FA sizes were all largely unchanged in Daam1/2-KO cells compared to controls (Fig. 6I-K). Nevertheless, our images suggested that the trailing edges of mutant cells on average exhibited larger FAs, indicative of potential changes in FA turnover. Thus, to assess FA dynamics, we transfected control and Daam1/2-KO cells with EGFP-paxillin and monitored adhesions by TIRF imaging during random migration on laminin (Movie 10). Temporal color-coded images, enabling comparison of FAs in cells over time, revealed that FAs in the double mutants were more stable than in control cells (Fig. 6L). Autocorrelation analysis of EGFP-paxillin fluorescence further showed that FA turnover was substantially slower in Daam1/2-KO cells, with quantification revealing a 57% increase in autocorrelation half-life compared to B16-F1 control (Fig. 6M, N). Consistently, both FA sliding velocity and disassembly rate in the double mutant were markedly impaired at the cell rear (Fig. 6O, P), whereas the FA assembly rate remained unchanged (Supplementary Fig. 15). Together, these findings suggest that perturbed disassembly of mature FAs at the rear impairs efficient rear retraction and thereby contributes substantially to the migration defects of Daam-deficient mutants.

### Daam, FMNL and mDia formins jointly regulate cortical rigidity and cytokinesis

**Fig. 7:**
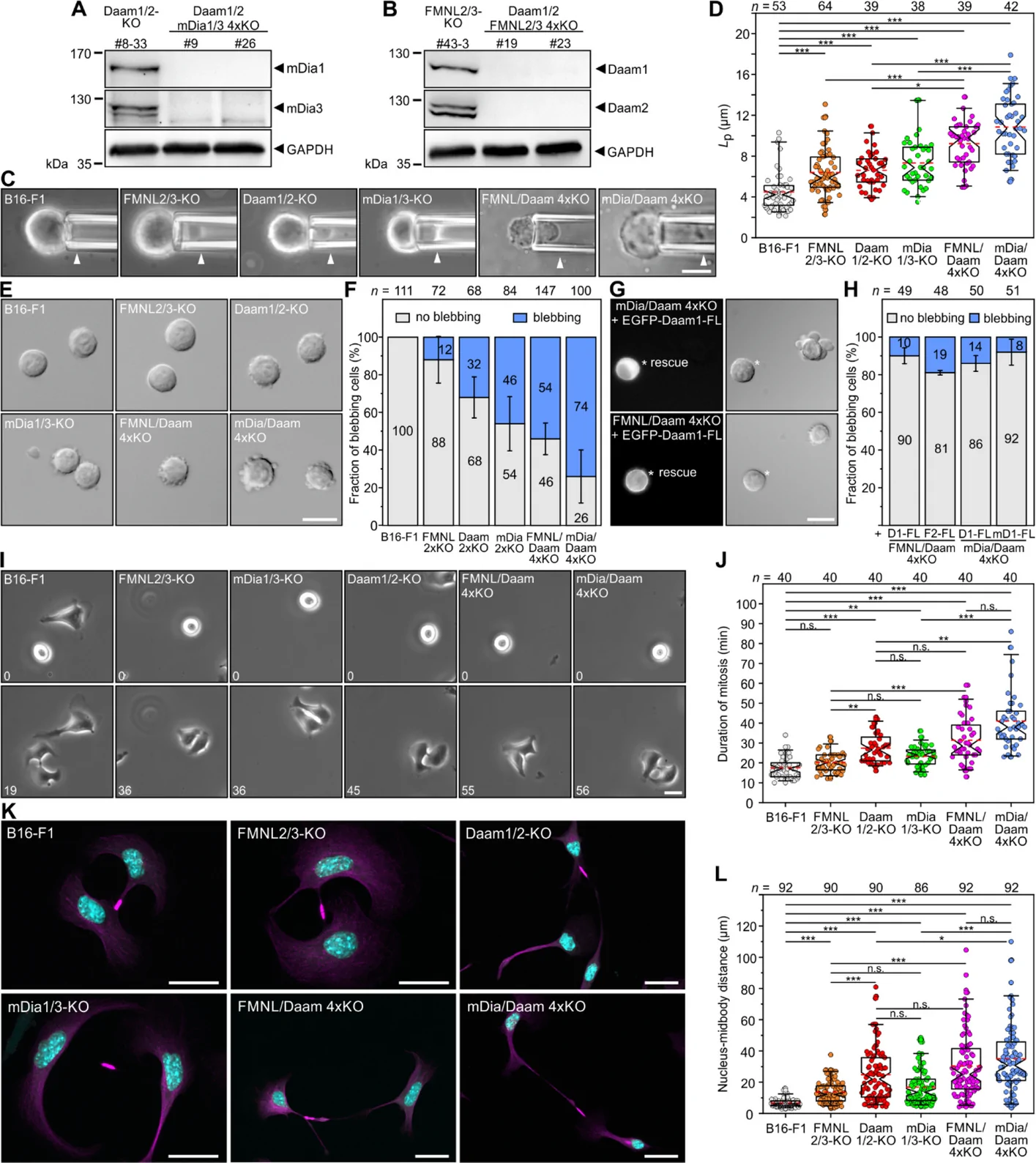
Loss of cortical formins impairs cortical integrity and cytokinesis. **A**, **B** Loss of mDia1 and mDia3 in Daam1/2-KO cells and of Daam1 and Daam2 in FMNL2/3-KO cells by CRISPR-Cas9 was confirmed by immunoblotting using specific antibodies. GAPDH was used as loading control. **C** Representative images of B16-F1 control and mutant cells as indicated from micropipette aspiration assays with white arrowheads indicating the initial indentation length (*L*p). Scale bar, 10 μm. **D** Quantification of *L*p-values in control and mutant cells determined by micropipette aspiration using a constant suction pressure of 50 Pa. **E** Representative differential interference images (DIC) of B16-F1 control and mutant cells on glass. **F** Quantification of blebbing. Note, most prominent blebbing in both quadruple mutants. **G** Reconstitution of both quadruple mutants by transient expression of full-length (FL) formins, shown here exemplarily for EGFP-tagged Daam1-FL. Corresponding fluorescence and DIC images are shown, and expressing cells are marked with an asterisk. **H** Quantification of blebbing in both 4xKO mutant cells either rescued by EGFP-tagged Daam1-FL (D1-FL), FMNL2-FL (F2-FL) or mDia1-FL (mD1-FL). **I** Still images from representative time-lapse movies displaying B16-F1 control and formin-KO cells during cytokinesis. **J** Quantification of the duration of cell division in control and mutant cells. **K** Representative images of B16-F1 and mutant cells stained for tubulin and nuclei. Brightest-point projection of confocal stacks. **L** Quantification of the midbody to nucleus distance. Scale bars in E, G, I and K, 20 µm. *P*-values in D, J and L were determined using Kruskal–Wallis ANOVA followed by Dunn’s multiple comparison test. Boxes in box plots indicate 50% (25 to 75%) and whiskers 90% (5 to 95%) of all measurements, with dashed red lines depicting the means. Medians are highlighted by indentation of boxes. *n*, number of cells analyzed. * *P* < 0.05, ** P < 0.01, *** *P* < 0.001, ns: not significant

To examine the potential cooperative role of cortical formins in regulating cortex mechanics in B16-F1 cells, we then disrupted mDia1 and mDia3 expression in Daam1/2-deficient cells as well as Daam1 and Daam2 expression in FMNL2/3-deficient cells using CRISPR/Cas9 technology. Respective protein loss in independent clonal lines of the resulting quadruple (4xKO) mutants was again confirmed by immunoblotting and TIDE or DNA sequencing analyses (Fig. 7A, B and Supplementary Fig. 16). To assess the viscoelastic properties of control and mutant cells, we performed micropipette aspiration assays (MPAs) on suspended cells at a constant suction pressure of 50 Pa, as shown in Fig. 7C. This enabled direct measurement of cortical rigidity and comparison with the previously published FMNL2/3- and mDia1/3-KO mutant cell lines [39, 48]. Initial indentation lengths (*L*p) were already increased in all FMNL2/3-, Daam1/2-, and mDia1/3-double mutants and were further elevated in both 4xKO cells, however, with mDia/Daam 4xKO cells displaying the strongest defect (Fig. 7D). These data indicate a markedly perturbed cell cortex in the quadruple mutants and support the notion of progressively increasing defects in cortical rigidity, likely depending on the specific activities of these formins. Notably, cortical defects are frequently associated with blebbing, characterized by blister-like protrusions of the plasma membrane [12, 76]. We therefore employed time-lapse differential interference contrast (DIC) microscopy to monitor the behavior of control and mutant cells after seeding on uncoated glass-bottom dishes (Fig. 7E and Movie 11). Unlike control cells, which showed no detectable blebbing, and FMNL2/3-KO cells, which displayed only minor blebbing defects (12%), approximately one third of Daam1/2-KO cells exhibited pronounced blebbing. This phenotype was further enhanced in cells lacking mDia1 and mDia3 (46%) and was most pronounced in the two 4xKO mutants, with blebbing observed in 54% of FMNL/Daam 4xKO cells and 74% of mDia/Daam 4xKO cells (Fig. 7F). The specificity of the phenotype was confirmed by rescue experiments, in which transient expression of EGFP-tagged mDia1-FL or Daam1-FL largely abolished blebbing in mDia/Daam 4xKO cells, and expression of FMNL2-FL or Daam1-FL rescued the blebbing phenotype in FMNL/Daam 4xKO cells (Fig. 7G, H and Movie 12). Together, these findings further support a cooperative role of these cortical formins in maintaining cell cortex integrity. Of note, besides their critical function in interphase cells, mDia1 and mDia3 have been also implicated in cytokinesis [88]. Thus, we then asked whether, or to what extent, mitotic cell division is impaired in the formin KO cell lines. To this end, we imaged the cells on laminin by time-lapse phase-contrast microscopy and determined the time from the onset of prometaphase until completion of cytokinesis (Fig. 7I and Movie 13). The duration of this process was only moderately increased in the double mutants but was substantially prolonged in both quadruple mutants, with Daam/mDia 4xKO cells again displaying the strongest phenotype, as evidenced by the longest delay in the completion of cell division (Fig. 7J). Unlike control cells, we further noticed that the daughter cells of the mutants did not separate rapidly after mitosis, but remained connected for quite a long time by a cytoplasmic bridge. To corroborate this observation, we stained fixed cells on laminin for microtubules and DNA (Fig. 7K), and quantified the distance between the midbody and the nucleus as a measure of perturbed cytofission. Notably, we found an incremental increase in the midbody-to-nucleus distance with 13.7 ± 7.0 µm in FMNL2/3-KO cells, 16.9 ± 11.0 µm in mDia1/3-KO cells, 24.8 ± 17.0 in Daam1/2-KO cells as well as 30.3 ± 20.9 µm in the FMNL/Daam 4xKO and 35.0 ± 21.6 µm in mDia/Daam 4xKO, compared to 6.7 ± 2.7 µm in B16-F1 controls (Fig. 7L). Combined, these observations reveal a critical cooperative role for FMNL-, mDia- and Daam-family formins in maintaining cortical function and supporting mitotic cell division.

### Combined loss of mDia1/3 and Daam1/2 augments NM2 activation and perturbs rear cortex function

**Fig. 8:**
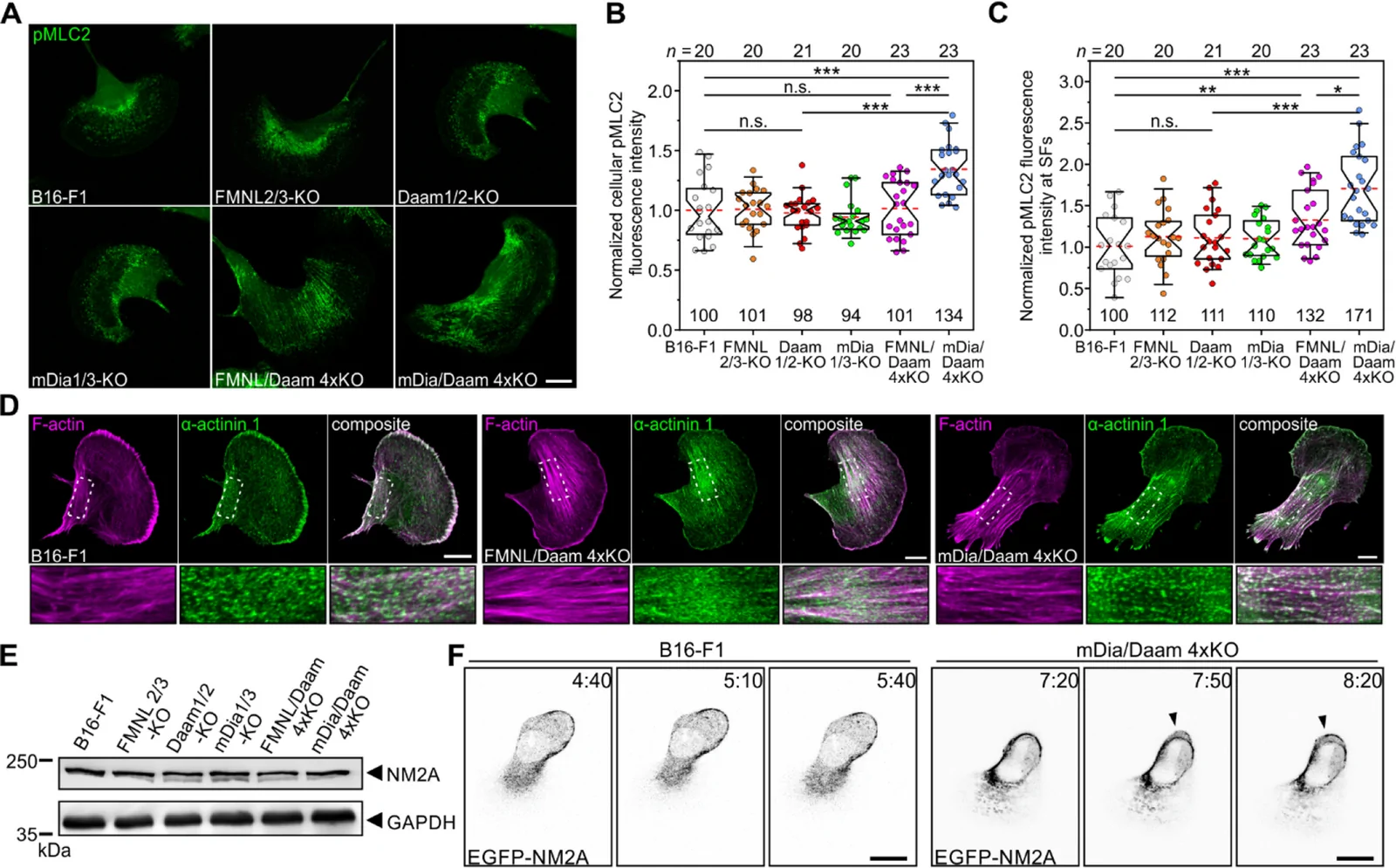
Combined loss of mDia1/3 and Daam1/2 enhances NM2 activation and results in rear-end blebbing. **A** Representative images of fixed B16-F1 and mutant cells stained for pMLC2. **B** Quantification of cellular pMLC2 intensity **C** Quantification of pMLC2 intensity at SFs. B, C Percentages at the bottom of graphs are shown to better illustrate the differences between cell lines. **D** Representative images of B16-F1, FMNL/Daam 4xKO, and mDia/Daam 4xKO cells stained for α-actinin. The F-actin cytoskeleton was visualized with phalloidin. Regions indicated by white rectangles are shown at higher magnification below. **E** Immunoblot depicting NM2A levels in B16-F1 and derived mutant cells. GAPDH served as a loading control.** F** Inverted still images from time-lapse movies of control and mDia/Daam 4xKO cells ectopically expressing EGFP-NM2A during migration on laminin. Confocal section about 2 µm above the substratum are shown. Time points are indicated in min:s. in the upper right corner. Black arrowheads highlight blebbing in the contractile rear cortex of the mDia/Daam 4xKO mutant. Scale bars in A, D and F, 10 µm. A and D, brightest-point projection of confocal stacks. *P*-values in B were calculated by one-way Anova whereas *P*-values in C were determined using Kruskal–Wallis ANOVA followed by Dunn’s multiple comparison test. Boxes in box plots indicate 50% (25 to 75%) and whiskers 90% (5 to 95%) of all measurements, with dashed red lines depicting the means. Medians are highlighted by indentation of boxes. *n*, number of cells analyzed. * *P* < 0.05, ** P < 0.01, *** *P* < 0.001, ns: not significant

DRFs and NM2 are tightly interconnected regulators of cortical actin dynamics and cellular contractility, with linear formin-elongated actin filaments providing the structural framework for contractile actomyosin networks [20]. This prompted us to examine whether loss of distinct formin subsets alters NM2 activation and organization. To this end, we first stained the formin mutant cell lines for phosphorylated myosin light chain 2 (pMLC2) as readout for active NM2 (Fig. 8A). pMLC2 accumulated at the cell rear and along filamentous structures spanning the cytoplasm, with the latter being particularly pronounced in both 4xKO cell lines. Nevertheless, among all analyzed mutants, only the mDia/Daam 4xKO exhibited a significant increase in total cellular pMLC2 levels (Fig. 8B), although enhanced pMLC2 accumulation at the filamentous network was observed in both 4xKO mutants (Fig. 8C), with this effect again being much more pronounced in the mDia/Daam quadruple mutant. Subsequent α-actinin-1 staining of B16-F1 control cells and both quadruple mutants revealed a periodic, striated pattern along actin filament bundles, confirming their identity as SFs (Fig. 8D). Consistent with enhanced NM2 activation at SFs in both quadruple mutants, and despite unchanged overall NM2A expression levels (Fig. 8E), we additionally found markedly increased accumulation of the tension marker zyxin at the SF-FA anchor sites in the mDia/Daam 4xKO. Together with increased FA area in both quadruple mutants (Supplementary Fig. 17), this indicates amplified strain on their SF-FA system [71, 76, 92]. Given that active NM2 drives actomyosin contractility and thereby increases intracellular hydrostatic pressure [17, 64], we finally monitored rear-cortex dynamics in EGFP-NM2A-expressing B16-F1 control cells and both 4xKO mutants migrating on laminin. In line with the immunofluorescence data obtained from fixed cells (Fig. 8A, C), live-cell imaging revealed a redistribution of NM2A to SFs during random 2D cell migration on laminin in both quadruple mutants (Movie 14). To better elucidate the consequences of this altered contractile organization and behavior for cortical integrity, we next analyzed confocal sections captured approximately 2 µm above the substratum to specifically visualize dynamics of the bulgy rear cortex in migrating cells. Strikingly, extensive and persistent blebbing was observed exclusively in mDia/Daam 4xKO cells, whereas wild-type, mDia1/3-KO and FMNL/Daam 4xKO cells maintained a smooth and stable rear cortex (Fig. 8F and Movie 15). Together, these observations revealed a profound mechanical weakness of the mDia/Daam-deficient cortex, which appears unable to withstand contractile forces generated by NM2 during trailing-edge retraction in migrating cells.

## Discussion

With this study, we aimed to define the complete inventory of DRFs contributing to the assembly of the contractile actin cortex in B16-F1 melanoma cells. Building on previous work, which identified mDia1 and mDia3 as key cortical formins [12, 48, 73, 76], we used a multilayered approach combining Y2H interaction studies with subsequent functional analyses to systematically screen candidates among the remaining DRFs for interactions with Rho-family GTPases implicated in contractility. This led to the identification of Daam1, Daam2, FMNL2, and FMNL3 in these pathways. Active variants of all four formins fused to EGFP accumulated prominently at the contractile rear cortex of polarized B16-F1 cells as well as NIH 3T3 fibroblasts, a localization that was also confirmed by antibody staining of the endogenous proteins. In case of Daam1, this is partly consistent with an earlier study likewise employing a self-generated polyclonal antibody raised against the C-terminal half of Daam1, which detected the protein at contractile SFs, the centrosome and cortical regions in COS-7 and U2OS cells [3]. By contrast, another study reported Daam1 accumulation within lamellipodia and along the shafts and tips of filopodia and microspikes at the leading edge of B16-F1 cells [38], highlighting an apparent discrepancy that may, at least in part, reflect the use of different antibodies and remains to be resolved in future studies. For FMNL2, rear cortical accumulation was also apparent in images from previous studies, although it was not further addressed at the time [9]. Our localization data, together with the RNA-Seq data showing prominent expression of mDia1/3, Daam1/2, and FMNL2/3 in B16-F1 cells, indicate that cortex regulation in these cells is not mediated by a single formin but rather safeguarded by the cooperative activity of multiple DRFs with overlapping functions, as previously observed in the *Dictyostelium* model system [48].

Earlier studies examining the interactions between various Rho-family GTPases and mammalian Daam1 using immunoprecipitations and GST pull-down assays, have yielded partly divergent or even conflicting results. While one study reported interactions of Daam1 with active RhoA and Cdc42, but not Rac1 [5], another found binding to active RhoA, RhoB, RhoC and RhoD, but not Rac1, Cdc42, RndI, Rif, RhoG, Tc10 and RhoJ [36]. Using a complementary Y2H-assay, we confirmed robust interactions of Daam1 with active RhoA, RhoB, RhoC, RhoD and Cdc42, and thus expanded the repertoire of interacting GTPases to active Rif and a weak interaction with Rac1. Daam2 displayed a comparable interaction profile, supporting the view that both Daam-family members are embedded in similar upstream signaling networks. Beyond confirming the Y2H data by pull-down experiments, the most compelling evidence for DRF regulation arises from the activation of the autoinhibited formin by an active GTPase. As full-length formins cannot be functionally purified from *E. coli*, we generated an autoinhibited “sandwich” complex by co-expressing the N- and C-terminal fragments of Daam1. Bulk pyrene assays conducted with this complex in the absence of PFN1 revealed that the Rho-family GTPases identified in the Y2H screen can also activate Daam1, thereby markedly enhancing nucleation relative to control. These findings parallel earlier work from the Higgs lab, which demonstrated that active RhoA, at least partially, releases mDia1 from autoinhibition using a similar bulk assay [47]. Interestingly, Higashi and colleagues previously reported that human Daam1 is activated by GTP-bound RhoA, RhoB, and RhoC, but not Rac1 or Cdc42, using glutathione-Sepharose beads coated with an autoinhibited Daam1 complex [36]. However, the inherent complexity of the employed cytosol-based assay, which revealed only rhodamine-phalloidin accumulation on the bead surface in static images, precluded detailed mechanistic interpretation. In contrast, we here report the first direct visualization of the release of Daam1 autoinhibition by active variants of identified GTPases in real time at the single filament level using TIRF imaging. In the presence of PFN1, formed filaments elongated at ~ 60 subunits per second, thus closely matching the rate observed for the unregulated Daam1-C fragment and thereby classifying Daam1 as a potent filament elongator akin to mDia1 [43]. Daam2-C exhibited similar biochemical activities, but acted as a slightly weaker elongator and a stronger nucleator compared to Daam1.

Because DRF activation in cells requires both GTPase binding and spatial coincidence with active upstream regulators, the rear-cortical localization of endogenous Daam1/2 suggests that their relevant activation in migrating B16-F1 cells is likely dominated by Rho-family GTPases enriched or active at the rear. RhoA signaling is well known to promote actomyosin contractility through ROCK and NM2 [53, 63], and recent work further supports a long-range interplay between Rho- and Rac-driven polarity modules during migration [6]. Our data are therefore consistent with a model in which Daam1/2 operate mainly downstream of RhoA, RhoB, RhoC, RhoD and potentially Rif to reinforce the contractile cortex in the rear. Although Rac1 and Cdc42 are classically associated with lamellipodia and filopodia at the cell front [70], their biochemical ability to activate Daam1 does not exclude context-dependent roles at the rear. In particular, Cdc42 has also been implicated in rear-associated signaling and contractility previously [56, 95]. Thus, it will be important to define the local GTPase activities that directly engage Daam-family formins in migrating cells in future work.

Similar to Daam1, the regulation of FMNL2 remained a matter of debate. One study reported on the co-crystallization of an N-terminal FMNL2 fragment (aa 1–379) in complex with active Cdc42, but found no detectable binding to active Rac1 using isothermal titration calorimetry (ITC) [45]. In contrast, another study employing siRNA-based screening and pull-down assays concluded that FMNL2 acts exclusively downstream of RhoC to drive amoeboid cell migration [41]. In subsequent work, the same group reported an additional context in which FMNL2 interacts with Rac1, i.e. during junctional actin assembly in epithelial cells [31, 32]. Our data largely reconcile these controversies. We detected prominent interactions of FMNL2 with active RhoA, RhoC, Rif, Rac1, and Cdc42 in the Y2H assay, and confirmed these findings in GST pull-down and activation assays. Analytical ultracentrifugation further showed that the apparent GTPase-binding properties of FMNL2 depend strongly on the N-terminal fragment analyzed. Interestingly, the shorter FMNL2-N2 construct, corresponding to the crystallized fragment used by Kühn and colleagues [45], bound Cdc42 with high affinity, while binding to RhoA and Rac1 was significantly weaker. By contrast, the longer FMNL2-N1 construct harboring the complete FH3 domain, exhibited more comparable binding to all tested GTPases. Thus, construct boundaries appear to be critical determinants of the observed FMNL2-GTPase interaction profile, explaining controversial results in these studies. In cells, this biochemical versatility is mirrored by the localization of FMNL2 to both the leading edge and rear cortex [9, 39], supporting its role as a dual-role formin, coordinating actin-based protrusion at the cell front and actomyosin-based contractility in the rear. Of note, GTPase binding alone does not necessarily determine the subcellular localization of a full-length formin. For instance, recruitment of mDia1 and mDia2 has been shown to critically depend on multiple binding modules, including a basic N-terminal stretch in addition to the GBD [30, 64, 65]. Similarly, in FMNL-family formins, blocking N-terminal myristoylation is sufficient to prevent membrane recruitment [9]. Thus, while GTPase binding is clearly relevant for regulation, it is generally not sufficient on its own to explain subcellular targeting. Notwithstanding this, we assume that DRFs such as FMNL2 are coincidently activated upon colocalization with their corresponding GTPases.

The phenotype of Daam1/2-deficient cells, together with our localization data, suggests that Daam-family formins primarily contribute to cortex-dependent polarity and rear-end dynamics rather than to filopodia or microspike formation at the cell front, despite an earlier study proposing cooperation between Daam1 and fascin in protrusion formation in B16-F1 cells [38]. Although independent studies support close proximity between Daam1 and fascin in cells [33, 57], there remains an apparent disconnect between their reported association and a functional role for Daam1 in maintaining filopodia integrity, as Daam1 depletion was subsequently reported to affect neither filopodia number and length nor fascin dynamics within these structures, although filopodia lifetime was slightly increased [57]. Consistent with these functional observations, we did not detect a direct interaction between fascin and Daam1, and more importantly our Daam1/2-deficient cells also formed microspikes with normal density, length, and fascin localization. Moreover, Arp2/3 complex, WAVE2 and VASP localization as well as lamellipodial F-actin content, lamellipodial width, actin network assembly and protrusion rates all remained essentially unchanged upon Daam1/2 loss. Thus, the reduced 2D migration of Daam1/2-KO cells cannot be explained by defective lamellipodial actin polymerization or impaired microspike assembly. Instead, the decreased protrusion persistence, the frequent formation of multiple fronts and the elongated cell morphology rather point to a defect in coupling protrusive activity with rear end retraction to enable effective cell translocation. In fact, similar to NIH 3T3 fibroblasts lacking mDia1 and mDia3 [76], Daam1/2-deficient B16-F1 mutant exhibited pronounced defects in FA turnover, although global FA number and size were not markedly altered. Since we did not observe direct accumulation of Daam1/2 at FAs in B16-F1 cells, our findings suggest that Daam-family formins are not required for FA formation in this cell type. Instead, the combined defects in cortical mechanics, rear retraction, and FA dynamics support a model in which loss of Daam-formin function indirectly affects FA disassembly through altered mechanical properties and force transmission at the cell rear. Interestingly, however, several previous studies have reported Daam1 localization to the cell cortex and contractile SFs [3, 12, 18, 51] which are mechanically coupled to FAs. Chua and colleagues additionally observed Daam1 accumulation at FAs in human U2OS cells, suggesting a more direct involvement of Daam1 in FA dynamics, at least in this cell type. Notably, Daam1 depletion in that study resulted in an elongated cell morphology, frequently accompanied by abnormally long tails. Consistently, the B16-F1 derived Daam1/2 double-knockout cells analyzed here also displayed a pronounced defect in rear-end retraction, a process known to be Rho-dependent [90], and frequently formed unusually long tethers that eventually ruptured, detached from the cell body, thereby generating cytoplasts (Fig. 6E, Movie 9). Most importantly, we directly demonstrated that combined loss of Daam-family formins compromises the rigidity of the viscoelastic cell cortex, analogous to the defects observed in FMNL- and mDia-family formin knockout mutants. However, the individual contributions of Daam1 and Daam2 cannot be fully resolved, as Daam2 was subsequently disrupted in the Daam1-KO background and most functional analyses were performed in the resulting double-KO cells. Similar to observations in *Dictyostelium* [48], B16-F1 double formin-null mutants displayed only intermediate defects in cortical function, whereas concurrent loss of multiple formins in both quadruple mutants caused much stronger defects in cortical rigidity, highlighting their functional cooperativity. While the mDia/Daam quadruple-knockout cells required the longest time to complete cytokinesis and frequently formed long tether-like structures between daughter cells due to inefficient separation, FMNL/Daam 4xKO cells showed only modest, statistically not significant increases in these parameters compared with the Daam1/2 double knockout. Interestingly, abscission during late cytokinesis has been reported to be PFN-dependent [11], supporting a critical role for formin-mediated actin filament elongation in this process. Moreover, both of the quadruple mutants also showed enhanced blebbing compared with the Daam1/2- and mDia1/3-double mutants, indicative of severe defects in cortical integrity. Remarkably, expression of EGFP-Daam1-FL, EGFP-mDia1-FL and FMNL2-FL-EGFP was sufficient to effectively suppress blebbing in both 4xKO mutants. This supports the idea that cortical function and rigidity depend primarily on the abundance of linear actin filaments in the cortex, rather than on filament generation by a specific formin. Consistent with this notion, FMNL2 and FMNL3 exhibit lower nucleation and elongation activities compared to mDia- and Daam-family formins, at least *in vitro* [9, 39], which may explain why the defects are less pronounced in the FMNL/Daam 4xKO than in the mDia/Daam 4xKO.

Finally, despite unchanged expression of NM2A in our collection of mutants, active NM2 is particularly upregulated in the Dia/Daam 4xKO. Because active NM2 generates contractile stresses and intracellular hydrostatic pressure that must be resisted by a mechanically resilient cortex [17, 20], this may further predispose this mutant to cortical failure. It is therefore not surprising that only the mDia/Daam 4xKO mutant exhibited prominent blebbing at the contractile rear cortex, a region where bleb formation is typically suppressed to maintain front-directed protrusion and drive effective migration in different cell types [64, 67]. As previously shown after loss of mDia1/3 in NIH 3T3 fibroblasts, this appears to redirect actomyosin-generated forces towards the SF-FA system [76], potentially supporting rear retraction in the absence of a fully functional contractile cortex. Collectively, these findings establish Daam1, Daam2, FMNL2, and FMNL3 as key contributors to cortical rigidity and mechanics, expanding the inventory of cortical formins beyond mDia1 and mDia3.

## Methods

### Constructs

For Y2H analysis, the cDNAs of murine Rho-family GTPases, RhoA (Uniprot ID:Q9QUI0), RhoB (Uniprot ID:P62746), RhoC (Uniprot ID:Q62159), RhoD (Uniprot ID:P97348), RhoF/Rif (Uniprot ID:Q8BYP3), Rac1 (Uniprot ID:P63001) and Cdc42 (Uniprot ID:P60766-1) all lacking their CAAX motifs were amplified by PCR from a B16-F1 cDNA library [60] and inserted into the EcoRI and SalI sites of plasmid pGBKT7 (Clontech). Respective constitutively-active and dominant-negative variants were generated by PCR using standard procedures to yield prey plasmids. The cDNAs encoding murine FHOD1-GBD/FH3 (Uniprot ID:Q6P9Q4, residues 53–462), FMNL2-GBD/FH3 (Uniprot ID:A2APV2-3, residues 23–469), FMNL3-GBD/FH3 (Uniprot ID:Q6ZPF4-1, residues 26–472), and Daam1-GBD/FH3 (Uniprot ID:Q8BPM0-2, residues 45–420) were amplified from the B16-F1 cDNA library and inserted into the BamHI and XhoI sites of plasmid pGADT7 (Clontech), and the amplified GBD/FH3 region of murine Daam2 (Uniprot ID:Q80U19, residues 40–420) was inserted into the XmaI and XhoI sites of pGADT7 to generate the bait plasmids.

For expression of constitutively-active and dominant-negative GTPases in *E. coli*, the coding sequences were amplified from the constructs mentioned above and inserted either into the BamHI and SalI sites (RhoA, RhoC, RhoD, Rac1, and Cdc42) or into the SalI and NotI sites (RhoB and Rif) of plasmid pGEX6-P-3 (GE Healthcare). The cDNA fragments encoding Daam1-C and Daam2-C were amplified from the B16-F1 library and inserted into the BamHI and SalI sites of plasmid pGEX-P-3. The amplified cDNAs encoding Daam1-N and Daam1-C of the complex were inserted into the SalI-NotI and MunI-XhoI sites of plasmid pETDuet-1 (Novagen), respectively. Fragments encoding FMNL2-N1 (residues 1–526) and FMNL2-N2 (residues 1–379) were amplified from plasmid pFMNL2-N1-EGFP [9] and inserted into the EcoRI and SalI sites of plasmid pGEX-6P-1. For co-expression of the autoinhibited FMNL2 complex, respective fragments (FMNL2-N1 and FMNL2-C encompassing residues 558–1092) were amplified from pFMNL2-EGFP and inserted into the SalI-NotI and FseI-XhoI sites of plasmid pETDuet-1, respectively. The plasmids for expression of GST-tagged fascin [15] and tag-free profilin I [14] were described previously. For expression of constructs in B16-F1 cells, cDNAs encoding constitutively-active Daam1ΔDAD (residues 1–1016,UniProt Q8BPM0-2) and Daam2ΔDAD (residues 1–1016) were amplified by PCR from a B16-F1 cDNA library. Notably, the obtained Daam2 cDNA sequence is identical to UniProt entry Q80U19-1, except that it lacks 48 residues (residues 652–699) corresponding to part of a predicted flexible loop within the FH2 domain. Subsequently, the PCR fragments were cleaved with BspEI-Asp718 or the XhoI-Acc65I, respectively, and inserted into the same sites of pEGFP-C1 (Clontech). Fidelity of generated plasmids was confirmed by DNA sequencing. For expression of EGFP-NM2A a DNA fragment containing the CMV promoter and the cDNA of EGFP from pEGFP-C1 (Clontech) was amplified by PCR and inserted into the AseI-XhoI sites of Myosin-IIA-GFP (Addgene plasmid #38,297). The following constructs were previously described: FMNL2-E272-EGFP and FMNL3-E275-EGFP [39], pEGFP-FHOD1ΔDAD [29], pEGFP-Rif-L77 [23], pEGFP-fascin [1], paxillin-EGFP [71], and pmCherry-C1-LifeAct [25]. pEGFP-human-β-actin was obtained from Clontech.

### Y2H assay

Protein–protein interactions were analyzed using the Matchmaker GAL4 Two-Hybrid System 3 (Clontech). AH109 yeast cells were co-transfected with the bait and prey vectors and grown on synthetic double-dropout agar lacking leucine and tryptophan at 30 °C for four days. Interactions were scored by growth of cells on triple-dropout (TD) or quadruple-dropout (QD) agar plates. TD plates lacked leucine, tryptophan and histidine, and were supplemented with 3 mM 3-amino-1,2,4-triazole to suppress the leaky HIS3-reporter gene as suggested by the manufacturer. QD plates lacked leucine, tryptophan, histidine, and adenine for highest stringency.

### Cell culture and transfection

B16-F1 mouse melanoma (ATCC CRL-6323) and derived cells were cultured at 37 °C and 5% CO_2_ in high-glucose DMEM culture medium (Lonza) supplemented with 1% penicillin–streptomycin (Biowest), 10% FBS (Biowest), and 2 mM UltraGlutamine (Lonza). After seeding onto 35 mm diameter wells (Sarstedt), cells were transfected with 1 μg plasmid DNA using JetPRIME transfection reagent (PolyPlus) according to the manufacturer's protocol.

### Genome editing by CRISPR/Cas9

The target DNA sequence of the murine Daam1 and Daam2 genes were pasted into the CRISPR/Cas9 design tool (https://cctop.cos.uni-heidelberg.de) to generate sgRNAs of 20 nucleotides with high-efficiency scores and minimal off targets. In case of Daam1, the targeting sequence 5’- ATAAAAGCACTGGGCGTTAT-3’in exon seven was used to cover all possible splice variants of the gene and for Daam2, the targeting sequence 5’-ATCGAAGCTTAGGCCGCTAC-3’in exon seven was used. Respective sequences were inserted into plasmid pSpCas9(BB)−2A-Puro(PX459)V2.0 (Addgene ID: 62,988) using BbsI [66]. CRISPR/Cas9 constructs were validated by DNA sequencing. For the generation of the quadruple mutants, previously described guides for mDia1 and 3 were used [48]. 24 h post transfection with the CRISPR constructs, the cells were selected in culture medium containing 2.5 µg/ml puromycin (Invivogen) for 4 days, and then cultivated for 24 h in the absence of puromycin. For isolation of clonal knockout cell lines, single cells were seeded into 96-well microtiter plates and expanded in sterile-filtered, pre-conditioned culture medium, composed of used and fresh medium at a ratio of 1:3. Subsequently, suitable fragments of about 500 bp spanning the respective target sites were amplified from genomic DNA, and clones analyzed by the TIDE sequence trace decomposition web tool (http://shinyapps.datacurators.nl/tide) [16] or DNA sequencing. Previously characterized cells lines included FMNL2/3 2x-KO clone #43–3 [39] and mDia1/3 2x-KO clone #1–20 [48].

### Protein purification

For expression of GST-fusion proteins, constructs were transformed into *E. coli* strain *Rossetta 2* (Novagen) and protein expression was induced with 750 µM isopropyl-β-D-thiogalactoside (IPTG, CarlRoth) at 21 °C for 14 h. The cells were then harvested and resuspended in lysis buffer containing 30 mM HEPES, pH 7.4, 150 mM NaCl, 2 mM EDTA, 2 mM benzamidine, 1 mM DTT, 5% (v/v) glycerol, 0.1 mM AEBSF (AppliChem) and 2 U/ml benzonase (Merck) and lysed by sonication. The same buffer was used to purify GST-fusions of Rho-family GTPases, except that EDTA was omitted. Fusion proteins were subsequently purified from bacterial extracts by affinity chromatography using Protino Glutathione Agarose 4B (Macherey–Nagel) employing standard procedures followed by optional cleavage of the GST-tag with PreScission protease (GE Healthcare). In a final polishing step, the proteins were further purified by size-exclusion chromatography (SEC) using HiLoad 26/75 Superdex, HiLoad 26/600 Superdex 200 or Superose 6 columns (GE Healthcare) controlled by an Äkta Purifier System. Constructs encoding the His-tagged autoinhibited formin complexes were transformed into *E. coli* strain *ArcticExpress* (Agilent Technologies), and protein expression was induced with 750 µM isopropyl-β-D-thiogalactoside at 12 °C for 14 h. Purification of His-tagged proteins with cOmplete His-Tag Purification Resin (Sigma) was performed according to the manufacturer’s instructions. Purified proteins were dialyzed against storage buffer (25 mM HEPES, pH 7.4, 150 mM NaCl, 1 mM DTT and 60% (v/v) glycerol) and stored at −20°C. Actin was purified from acetone powder of rabbit skeletal muscle as described previously [83]. Fractions were labelled on Cys374 with Atto488-maleimide (Atto-tec) and stored in G-Buffer (5 mM Tris/HCl, pH 8.0, 0.2 mM ATP, 0.5 mM DTT, 0.2 mM CaCl_2_, 0.1 mg/ml NaN_3_).

### Antibodies

Polyclonal antibodies against Daam1-C and Daam2-C were raised in female New Zealand White rabbits using the corresponding recombinant proteins described above as antigens, following standard procedures. The immunization of rabbits for polyclonal antibody production was performed in accordance with national guidelines for the care and use of laboratory animals and was approved by the Hannover Medical School Institutional Animal Care Facility and the Lower Saxony State Office for Consumer Protection and Food Safety (LAVES) under application number 18A255 to J.F. The following primary antibodies were used: rabbit anti-Daam1 (this study, 1:1000), rabbit anti-Daam2 (this study, 1:1000), rabbit anti-mDia1 (1:1000) and rabbit anti-mDia3 (1:000) [48], rabbit anti-FMNL2 (1:1000) and rabbit anti-FMNL3 (1:1000) [34], rabbit anti-VASP (1:1000) and rabbit anti-WAVE2 (1:1000) [23], mouse anti-Arp3 (1:1000,A5979, Sigma), mouse anti-α-tubulin (Merck, MAB1864, 1:500), mouse anti-fascin (undiluted hybridoma supernatant of clone 5E2) [23], rabbit anti-phospho-myosin light chain 2 (Ser19) (Cell Signaling, 3671S, 1:200), rabbit anti-NM2A (BioLegend, 909,801, 1:500) mouse anti-zyxin (Santa Cruz, sc-136128, 1:200), mouse anti-glyceraldehyde-3-phosphate dehydrogenase (GAPDH) (Merck, CB1001, 1:1000), and mouse anti-6x-His (Abcam, clone ab18184, 1:200). Primary antibodies in immunoblots were visualized with phosphatase-coupled anti-mouse (Dianova, 115–055–062, 1:1000) or anti-rabbit antibodies (Dianova, 115–055–144, 1:1000). Primary antibodies in immunohistochemistry were visualized with polyclonal Alexa488-conjugated goat-anti-rabbit (Thermo Fisher Scientific, A-11034, AB_2576217, 1:1000) or goat-anti-mouse antibodies (Thermo Fisher Scientific, A-11029, AB_2534088, 1:1000).

### Immunoblotting

For preparation of total cell lysates, cells were grown to confluency and trypsinized. Cell pellets were washed twice with ice-cold PBS and resuspended in ice-cold RIPA buffer (50 mM Tris/HCl, pH 8.0, 150 mM NaCl, 0.5% sodium deoxycholate, 1.0% Triton X-100, 0.1% sodium dodecyl sulfate) supplemented with 5 mM benzamidine, 0.1 mM AEBSF (Applichem), and benzonase (Merck). Suspensions were incubated for 1 h at 4 °C on a wheel rotator. Lysates were then clarified by centrifugation for 5 min at 16,000 × g, and the supernatant was mixed 1:1 with 3 × SDS loading buffer. After SDS-PAGE, proteins were transferred onto nitrocellulose membranes (Hypermol), blocked in NCP buffer (10 mM Tris/HCl, pH 8.0, 150 mM NaCl, 0.02% NaN₃, 0.05% Tween-20) containing 4% bovine serum albumin (BSA), and incubated overnight with primary antibodies diluted in NCP. After washing and incubation with secondary, phosphatase-conjugated antibodies, membranes were developed using 20 mg/ml 5-bromo-4-chloro-3-indolyl phosphate p-toluidine (BCIP) in NaHCO₃, pH 10.0.

### Pyrene assays

Recombinant Daam1/2-C or the autoinhibited Daam1 complex, in absence or presence of respective Rho-family GTPases, were diluted in 10 × KMEI buffer (100 mM imidazole, pH 7.0, 500 mM KCl, 10 mM MgCl_2_, 10 mM EGTA) to reach a final concentration of 1.18 × KMEI. Anti-foaming solution Extran AP33 (Merck) was added to a final concentration of 0.05% to reduce surface tension. Subsequently, 170 μl aliquots of the mixtures were placed in black, non-treated, 96-well microtiter plates (Brand GmbH). Actin polymerization was initiated by injecting 30 µl of 13.33 µM G-actin (5% pyrene-labeled) in G-buffer into the protein mixture using the automated dispenser of a Synergy 4 fluorescence microplate reader (BioTek), yielding final concentrations of 2 µM actin in 1 × KMEI and the concentrations of test proteins as indicated. After a 2 s mixing step, polymerization was monitored for 20 min by measuring pyrene-actin fluorescence at 364 nm excitation and 407 nm emission.

### Preparation of lipid vesicles

1,2-Dioleoyl-sn-glycero-3-phosphocholine (DOPC) in chloroform was obtained from Avanti Polar Lipids. Unilamellar lipid vesicles composed of DOPC were prepared by extrusion using an Avanti Mini-Extruder (Avanti Polar Lipids). The solvent from DOPC stock solutions was evaporated under a stream of dry nitrogen for 1 h. The dried lipids were resuspended in unilamellar vesicle buffer (20 mM HEPES, pH 7.4, 150 mM NaCl, 5 mM KCl, 3 mM MgCl₂, 5 mM β-mercaptoethanol,) at a total lipid concentration of 2 mg/ml, sonicated for 3 min using a tapered microtip, and then extruded 15 times through a 100 nm polycarbonate membrane (Avanti Polar Lipids) to obtain small unilamellar vesicles.

### *In vitro* TIRF microscopy

Glass coverslips were cleaned with 50 °C, bidistilled water (ddH_2_O) containing 2% (v/v) Hellmanex III for 15 min and then rinsed with ddH_2_O, followed by equivalent steps using 1 M KOH, 1 M HCl, and 70% ethanol, respectively. The coverslips were then dried and treated with room air plasma using an expanded table-top plasma cleaner (Harrick Plasma) for 30 min. Two-channel TIRF flow chambers were prepared by attaching the cleaned coverslips to glass slides using three small stripes of double-sided tape. Prior to the experiments, the channels were first filled with 20 μl of the lipid vesicle solution to form supported lipid bilayers on the solid glass surface within 30 min and then rinsed twice with 1 × KMEI buffer supplemented with 1% BSA. Reactions in TIRF assays contained 1 μM actin (spiked with 20% Atto488-conjugated monomers). For the experiments, Ca^2+^-G-ATP-actin was converted to Mg^2+^-G-ATP-actin by addition of 10 × magnesium-exchange buffer (1 mM MgCl_2_ and 10 mM EGTA). Polymerization experiments were performed in TIRF buffer containing 10 mM imidazole, pH 7.4, 50 mM KCl, 1 mM MgCl_2_, 1 mM EGTA, 0.2 mM ATP, 10 mM DTT, 15 mM glucose, catalase (20 μg/ml), glucose oxidase (100 μg/ml), and 0.5% methyl-cellulose (4000 cP). TIRF assays were initiated by adding ATP-G-actin and introducing the mixtures into preassembled flow chambers. Images were acquired on a Nikon Eclipse TI-E inverted microscope equipped with a CFI Apochromat TIRF 100x/1.49 NA oil-immersion objective and the 488 nm laser line. Images were recorded at 5 s intervals with 70 ms exposure using an iXon3 897 EMCCD camera (Andor) for at least 10 min. The pixel size corresponded to 0.159 µm. The elongation rates of growing actin filaments were determined by manually tracking their length over time using ImageJ software [75]. At least 13 filaments were measured from at least three independent movies per condition. For comparison of nucleation rates, the number of filaments formed over time was determined from at least three independent movies per condition.

### Low-speed sedimentation assay

For low-speed sedimentation assays, G-actin and Daam1-C were cleared for 60 min at 4 °C at 100,000 × g in a Beckman Optima TL-100 ultracentrifuge, and the reaction mixtures then incubated for 2 h at room temperature in 1 × KMEI buffer. Subsequently, the samples were centrifuged for 30 min at 4 °C and 17,000 × g in an Eppendorf table top centrifuge, and the pellets and supernatants brought to the original volume in SDS sample buffer. Pellet and supernatant fractions were analyzed by SDS-PAGE and Coomassie Blue staining to assess protein distribution.

### Pull-down assays

For GST pull-down assays, 100 µg of purified GST fusion proteins were bound to glutathione-agarose 4B beads (Macherey–Nagel) in pull-down buffer (1 × PBS, pH 7.4, 5 mM β-mercaptoethanol, 5 mM MgCl₂) by incubation on a wheel rotator for 1 h at 4 °C. Prior to the binding assay purified GST-GTPases were incubated with either 1 mM GTP or 1 mM GDP in pull-down buffer for 1 h on a wheel rotator at 4 °C. For binding experiments, the beads were washed five times with pull-down buffer and incubated with 100 μg each of respective test proteins in 1 ml of pull-down buffer. After 1 h of incubation at 4 °C on a wheel rotator, the beads were sedimented, washed five times with pull-down buffer, the bound proteins eluted with SDS sample buffer and then analyzed by SDS-PAGE.

### Analytical ultracentrifugation

Sedimentation velocity runs were performed in an Optima AUC analytical ultracentrifuge (Beckman Coulter) using an An-50 Ti rotor at 20°C. Concentration profiles were measured with absorption scanning optics at 280 nm using 3 or 12 mm standard double-sector centerpieces filled with 100 μl or 400 μl samples, respectively. Interaction experiments between FMNL2-N1 and the constitutively-active, GTP-saturated GTPase variants Cdc42-L61, Rac1-L61, and RhoA-L63 were conducted in a buffer containing 20 mM HEPES, pH 7.4, 150 mM NaCl, 1 mM MgCl₂ and 5 mM β-mercaptoethanol at 40,000 rpm. Due to the lower solubility of the shorter FMNL2 variant FMNL2-N2, interaction experiments were performed in 10 mM Tris/HCl, pH 8.0, 200 mM NaCl, 1 mM MgCl_2_, 5 mM β-mercaptoethanol and at 50,000 rpm. After mixing, all samples were allowed to equilibrate for 4 h at 20 °C prior to sedimentation analysis. Buffer density, viscosity and partial specific volumes were calculated from buffer and amino-acid composition using SEDNTERP3 [58]. Protein concentrations were determined spectrophotometrically using extinction coefficients at 280 nm calculated from amino-acid composition [55] and are given in monomers throughout the text. The extinction coefficient of GTP at 280 nm was determined spectrophotometrically to be 7900 M^−1^·cm^−1^ by referencing the manufacturer’s reported maximum absorption at 253 nm (ε_253nm_ = 13,700 M^−1^·cm^−1^, Thermo Scientific). This value was used to calculate the extinction coefficients of the GTPase/GTP complexes at 280 nm, assuming a 1:1 stoichiometry. Data analysis was performed using a model for diffusion-deconvoluted differential sedimentation coefficient distributions (continuous *c*(*s*) distributions) implemented in SEDFIT [79]. For the interactions of FMNL2-N2 and the GTPase/GTP complexes, signal-weighted average s-values, *s*_w_, were obtained by integrating the *c*(*s*) distributions in the s-value range where free proteins and complexes were observed using the program GUSSI [13]. Dissociation constants (K_*D*_) were determined by fitting an A + B ⇌ AB hetero-association model to the *s*_w_ isotherms using SEDPHAT [94].

### Immunofluorescence

For immunofluorescence labeling, cells were seeded on laminin-coated glass coverslips (25 µg/ml) (Ibidi) for 2 h and then fixed for 20 min in pre-warmed PBS, pH 7.4 supplemented with 4% paraformaldehyde (PFA) and 0.06% picric acid. Subsequently, the specimens were washed three times with PBS supplemented with 100 mM glycine, then permeabilized with 0.05% Triton X-100 in PBS for 45 s and blocked for 30 min with PBG (PBS supplemented with 0.045% cold fish gelatin and 0.5% BSA). For staining of endogenous formins, the cells seeded on laminin were fixed with 10% trichloroacetic acid (TCA) at 0 °C for 15 min. For fascin staining, the cells were fixed in methanol at −20°C for 10 min followed by step-wise rehydration. After blocking, the specimens were incubated with primary antibodies diluted in PBG overnight at room temperature. This was followed by washing fixed cells three times with PBG and incubation with secondary antibodies for at least 2 h. The EGFP signal was enhanced with Alexa488-conjugated nanobodies [59]. Atto550-phalloidin (Atto-Tec, AD550-8, 1:250) was used for visualization of filamentous actin and DNA was visualized with 4′,6-diamidin-2-phenylindol-dihydrochlorid (Sigma). The specimens were mounted in Mowiol (Sigma). Images of fixed cells were captured with an inverted Olympus XI-81 microscope equipped with an UPlan FI 100x/1.30 NA oil immersion objective or an UPlan FL 40x/0.75NA objective or a confocal Zeiss LSM 980 microscope with Airyscan 2 equipped with a Plan-Apochromat 63x/1.4 NA oil DIC objective. Image analysis was performed using the Fiji image processing software package [74]. Fluorescence intensities of antibody and phalloidin stainings were measured from images recorded at identical settings. Background fluorescence intensities were measured in regions outside of cells.

### Live cell imaging

Time-lapse imaging of cells was performed with an inverted Olympus XI-81 microscope (Olympus) driven by Metamorph software (Molecular Devices) and equipped with objectives specified below and a CoolSnap EZ camera (Photometrics). Unless stated otherwise, cells were seeded on laminin-coated 35-mm glass bottom dishes and allowed to adhere for at least 2 h. Prior to experiment, the medium was supplemented with 25 mM HEPES, pH 7.0, to compensate for the lack of CO_2_ during imaging in an Ibidi Heating System at 37 °C. For random motility assays, B16-F1 cells and derived KO-clones were seeded at low densities, allowed to adhere and then recorded by time-lapse phase-contrast imaging at 60 s intervals for 3 h using an Uplan FL N 4x/0.13NA objective (Olympus). Single cell tracking was performed in ImageJ using the MTrackJ plugin. Analysis of cell speed and MSD were performed in Microsoft Excel using a customized macro [49]. Cells that contacted other cells or divided were excluded from analyses. Lamellipodial protrusions were recorded at 5 s intervals for 10 min using an Olympus Uplan FL 100x/1.30NA objective. Protrusion rates of advancing lamellipodia assessed over a time period of 2.5 min were quantified by measuring the distance of the protruding front traveled over time. Lamellipodial persistence of randomly migrating B16-F1 wild-type and derived cells on laminin was determined by phase-contrast time-lapse microscopy using an Olympus Uplan FL N 4x/0.13NA objective and a frame rate of one frame per minute. For EGFP-fascin recordings, transfected cells were treated as described above and then imaged with an Olympus Plan FI 100x/1.30 NA oil immersion objective for 15 min at 10 s intervals. To monitor morphology and cytokinesis, the cells were imaged with an Olympus LUCPlan FL N 20x/0.45 NA objective for 60 min at 15 s intervals, or up to 12 h at 1 min intervals, respectively. For quantifying the duration of cytokinesis, the start was defined as the time point at which the cell became fully rounded, and the end as the moment when daughter cells had fully separated. For assessment of blebbing, live-cell DIC imaging was performed on freshly trypsinized cells plated on untreated glass-bottom dishes using an Olympus UPlan FL N 40x/0.75 P objective, condenser prism, and Nomarski/DIC optics for 5 min at 10 s intervals.

### Determination of actin assembly rates in lamellipodia

For quantification of lamellipodial actin polymerization rates, wild-type and mutant cells transiently expressing EGFP-β-actin were seeded on laminin-coated 35-mm glass bottom dishes (Ibidi) and maintained in growth medium for 2 h. Subsequently, the cells were recorded by time-lapse, phase-contrast imaging at 1.5 s intervals for 2 min using a Zeiss LSM 980 with Airyscan 2 equipped with a CO_2_ incubation system at 37 °C and a Plan-Apochromat 63x/1.46 NA oil DIC objective using a 488 nm laser. Emitted light was detected in the wavelength range of 490 to 649 nm. Three frames after the start of image acquisition, a region of interest (ROI) within the protruding lamellipodium was photobleached using the 488 nm laser at maximum power, with a 1 ms pixel dwell time, followed by quantification of fluorescence recovery in the lamellipodial actin network over time using Fiji. To this end, kymographs were generated from time-lapse line scans oriented perpendicular to the leading edge within the photobleached region, enabling independent quantification of protrusion and retrograde actin flow rates. The rate of actin polymerization within the lamellipodium was calculated as the sum of these two parameters.

### Quantification of FA parameters

For quantification of FA parameters, B16-F1 cells and derived mutants were pre-extracted with 0.3% Triton X-100 in 2% PFA in PBS for 30 s and then fixed for an additional 20 min in 4% PFA and 0.06% picric acid in PBS. Subsequently, the cells were washed, blocked with PBG as described above and stained for vinculin. TIRF images captured at identical settings were then analyzed using the Focal Adhesion Analysis Server [7], applying a detection threshold of 4 and a minimal adhesion size of 5 pixels. The extracted values for FA intensity, size, and number of adhesions per cell were subsequently analyzed in Microsoft Excel. To quantify zyxin localization at FAs, brightest-point-projections of confocal stacks from zyxin-stained cells were subjected to background subtraction using a rolling ball radius of 50 pixels. Subsequently, FAs with a minimum area of 0.1 μm^2^ were segmented, and the total FA area, normalized to the respective cell area. Mean zyxin intensity at FAs were quantified using a custom semi-automated ImageJ macro [34].

### Analysis of FA dynamics

B16-F1 cells and derived mutants transiently expressing EGFP-paxillin were seeded on laminin-coated 35-mm glass bottom dishes for 2 h, and then the medium was supplemented with 25 mM HEPES, pH 7.0. For analysis of FA dynamics, cells were imaged by time-lapse TIRF microscopy on an Olympus IX83 inverted microscope equipped with an UPlanApo 60x/1.50 Oil HR TIRF objective and an incubation system at 37 °C, acquiring images every 15 s for 15 min using the 488 nm laser line. To evaluate overall FA turnover, autocorrelation analysis was performed as previously described [93]. To this end, a mask of the EGFP-paxillin fluorescence in the first frame was generated by manually thresholding the image. Then, the sum of the pixel intensities within the defined mask for every second frame of the movie was normalized to the corresponding sum of the intensities in the first frame. This autocorrelation metric reflects turnover arising from changes in adhesion area, position, and intensity, rather than from the exchange of proteins between FAs and the cytosol to assess FA disassembly. Therefore, this approach allows assessment of the autocorrelation index as a measure of FA dynamics, where a value of one denotes complete stability and a value of zero indicates total loss of adhesions. To quantitatively compare FA turnover, the half-life of the autocorrelation function for each cell was estimated by linear interpolation between the two time points flanking the autocorrelation value of 0.5. To analyze FA sliding velocity, individual FAs at the cell rear were manually tracked using the MTrackJ plugin in ImageJ from the onset of sliding, initiated by cell body retraction, until their complete disassembly. FA assembly and disassembly rates were analyzed in Fiji using a modified version of the approach described previously [85]. Briefly, ROIs were manually placed over individual paxillin-positive FAs that were either assembling at the front or disassembling at the rear during the imaging period, and adjusted over time as needed to track adhesion dynamics. Background ROIs were generated by repositioning the original time-encoded ROIs to adjacent regions within the cell that lacked FAs. The fluorescence intensity within the ROIs was measured over time and corrected by subtracting background intensity values. Disassembly rate constants k_dis_ were calculated by fitting the fluorescence intensities of disassembling FAs over time to the exponential decay function.

$${I}_{\mathrm{d}\mathrm{i}\mathrm{s}\mathrm{a}\mathrm{s}\mathrm{s}\mathrm{e}\mathrm{m}\mathrm{b}\mathrm{l}\mathrm{y}}(t)={{A}_{0}+f}_{0}{\mathrm{e}}^{-{k}_{\mathrm{d}\mathrm{i}\mathrm{s}} t}$$

using the Solver add-in in Microsoft Excel by minimizing the sum of squares of the residuals with the offset *A*_0_, the amplitude* f*_0_ and *k*_dis_ as variables. The assembly rate constants *k*_ass_ were inferred by fitting the fluorescence intensities of assembling FAs over time to the logistic growth function.

$${I}_{\mathrm{a}\mathrm{s}\mathrm{s}\mathrm{e}\mathrm{m}\mathrm{b}\mathrm{l}\mathrm{y}}(t)=\frac{{f}_{\mathrm{m}\mathrm{a}\mathrm{x}}}{1+{\mathrm{e}}^{-{k}_{\mathrm{a}\mathrm{s}\mathrm{s}}(t-{t}_{{}^{1}/_{2}})}}$$

with the maximum fluorescence intensity *f*_max_, the time at half-maximum *t*_1/2_, and the rate constant *k*_ass_ as variables.

### Micropipette aspiration assay

Micropipette aspiration was performed as described previously [64]. Briefly, a measurement chamber with one open side was filled with 1 ml of PBS and mounted on a stage of an inverted Axiovert 200 microscope (Zeiss) equipped with an LD Achroplan 40x/0.6 objective (Zeiss). A BSA-coated glass micropipette with an inner diameter of 5.1 ± 0.9 μm was filled with PBS buffer and positioned into the chamber using an SM 3.25 micromanipulator (Märzhäuser). Aspiration pressure was applied and controlled using a height-adjustable water reservoir. The reference pressure (0 Pa) was calibrated by monitoring the motion of 2 μm polystyrene beads (Polysciences, 19814–15) in the pipette. After setting the pressure difference to 50 Pa, 50 μl of cell suspension was carefully injected into the chamber. Before cell adhesion on the glass substrate, only cells from the suspension were gently aspirated. The aspiration process was recorded with an AxioCam MRm camera (Zeiss) using ZEN core software (version 3.9) (Zeiss). Aspiration length (*L*p) was determined using Fiji software.

### RNA preparation-, sequencing- and data processing

For RNA sequencing experiments, B16-F1 mouse melanoma cells were seeded into 60 mm tissue culture-treated dishes (Sarstedt) and grown to approximately 90% confluency. Cells were then harvested on ice, and total RNA was extracted using the NucleoSpin® RNA Plus kit (Macherey–Nagel), following the manufacturer’s instructions, including a DNA digestion step using the rDNase Set (Macherey–Nagel). RNA extractions were carried out on three separate days. The purified RNA was eluted in RNase-free water, snap-frozen in liquid nitrogen and stored at −80°C. For RNA sequencing and data processing, the quality and integrity of total RNA were assessed using the Agilent 2100 Bioanalyzer (Agilent Technologies). RNA-seq libraries were prepared from 500 ng of total RNA with the NEBNext® Ultra™ II Directional RNA Library Prep Kit for Illumina® (New England BioLabs) in combination with the NEBNext Poly(A) mRNA Magnetic Isolation Module, following the manufacturer’s instructions. Libraries were sequenced on the Illumina NovaSeq X Plus platform using the NovaSeq X Plus 1.6B Reagent Kit (300 cycles, paired-end run), generating on average 3 × 10⁷ reads per RNA sample. FASTQ files were subjected to quality control with FastQC (www.bioinformatics.babraham.ac.uk/projects/fastqc). Prior to alignment, raw reads were trimmed for base-call quality and adapter contamination using fastq-mcf; reads shorter than 15 bp were discarded. Trimmed reads were aligned to the reference genome (Mus musculus GRCm39.109) using the open-source aligner STAR [26] with parameters as specified in the log files. Feature counting and read summarization were performed with Rsubread (v2.16.1), followed by annotation with biomaRt (v2.58.2). Data preprocessing and normalization were carried out using edgeR (v4.0.16), applying the trimmed mean of M-values (TMM) method [68].

### Quantification and statistical analysis

Quantitative experiments were performed at least in triplicates to avoid any potential environmental bias or unintentional error. The number of independent biological replicates as well as the number of events quantified for microscopy analysis is indicated in respective figure legends. Raw data were processed in Excel (Microsoft). No samples or data points were excluded from analysis. Statistical analyses were performed with Origin 2021 (OriginLab). All datasets were tested for normality by the Shapiro–Wilk test. Statistical differences between normally distributed data sets of two groups were determined by Student's t-test and not normally distributed datasets of two groups by the nonparametric Mann-WhitneyU rank sum test. For comparison of more than two groups, statistical significance of normally distributed data was examined by one-way analysis of variance (ANOVA) and Tukey’s multiple comparison test. In case of not normally distributed data, the nonparametric Kruskal–Wallis ANOVA and Dunn’s multiple comparison test were used. Statistical differences were defined as *P < 0.05, **P < 0.01, ***P < 0.001 as well as n.s., not significant, and are displayed and stated in figures and figure legends, respectively. All values are shown as means ± SD or means ± SEM. Superplots were generated as previously described [50].

## Supplementary Information

Supplementary Material 1. Supplementary Fig. 1. FHOD1 fails to interact with Rho-GTPases in the Y2H assay but drives the formation of SFs in B16-F1 cells.Presumably due to improper folding, no interactions were observed with the FHOD1-GBD/FH3 in the Y2H screen using either constitutively-activeor dominant-negativevariants of the tested Rho-family GTPases as bait. Protein-protein interactions were monitored by growth on selective triple-dropoutand quadruple-dropoutmedia. AD, GAL4-activation domain; BD, GAL4-DNA-binding domain; aa, amino-acid residues.Representative image of a B16-F1 cell transiently expressing constitutively-active EGFP-FHOD1Δ DAD lacking its C-terminal DAD domain. The F-actin cytoskeleton was visualized with phalloidin. Scale bar, 10 µm. Supplementary Fig. 2. Specificity of Daam polyclonal antibodies and subcellular localization of Daam1/2 and FMNL2/3 in B16-F1.Lysates from untransfected B16-F1 control cells and cells transiently expressing EGFP-Daam1-FL or EGFP-Daam2-FL were analyzed by immunoblotting using Daam1- or Daam2-specific polyclonal antibodies. Both antibodies detected the endogenous Daam proteins at approximately 120 kDa as well as their corresponding EGFP-fusion proteins, confirming antibody specificity.Representative images of B16-F1 wild-type cells and derived double mutants fixed with TCA and stained with the indicated antibodies. Scale bar, 10 µm. Supplementary Fig. 3. Daam- and FMNL-family formins also localize to the contractile cell cortex in NIH 3T3 cells.Representative images of NIH 3T3 cells ectopically expressing the indicated EGFP-tagged constitutively-active variants of Daam- and FMNL-family formins. Cells migrating on fibronectin were fixed with PFA, and the F-actin cytoskeleton was visualized with phalloidin.Representative images of NIH 3T3 fibroblasts on fibronectin, fixed with TCA, and stained with the polyclonal antibodies for endogenous proteins as indicated.Scale bars, 10 µm. Supplementary Fig. 4. RNA-Seq analysis reveals formin gene expression in B16-F1 cells. RNA-Seq libraries were sequenced and subjected to quality control prior to preprocessing and normalization using edgeRwith the trimmed mean of M-valuesmethod. Expression is shown as linear counts per million for each formin. Analysis of all 15 formins showed abundant expression of FMN1, mDia1/2, Daam1/2, and FMNL2/3, at least at the RNA level, whereas INF2, mDia3 and FHOD1 were expressed at comparatively low levels. The remaining formins appear not to be expressed in this cell type. The data represent arithmetic mean ± SD from three independent experiments. Supplementary Fig. 5. Schematic illustration of constructs used for biochemical characterization. Constitutively-active and dominant-negative GTPases were initially purified as GST-fusion proteins. One fraction was retained as GST-tagged protein for GST pull-down assays, whereas the GST tag was removed from the remaining fraction by cleavage with PreScission protease to obtain tag-free constructs, which were subsequently used in pyrene-actin polymerization and TIRF assays. Daam1/2-C and FMNL2-N1/N2 constructs were likewise initially purified as GST-fusion proteins, followed by GST-tag removal and further purification before use in biochemical experiments. Autoinhibited Daam1 and FMNL2 sandwich complexes were purified by Ni-NTA affinity chromatography using a His-tag fused to the respective N-terminal fragments, whereas the corresponding C-terminal fragments remained untagged. Numbers correspond to the respective terminal amino acids. Abbreviations: DAD, Diaphanous-autoinhibitory domain; GBD, GTPase-binding domain; FH1, formin homology domain1; FH2, formin homology domain2; FH3, formin homology domain 3; Domain architecture is not drawn to scale. Supplementary Fig. 6. Daam-family formins promote concentration-dependent actin assembly in pyrene-actin polymerization assays. Reactions contained 2 µM actinand the indicated concentrations of tag-free Daam1-Cor Daam2-C, respectively. Curves represent mean values from three independent measurements. SD values were omitted for clarity. Supplementary Fig. 7. Daam2-C does not exhibit actin filament bundling activity.Input samplesand low-speed sedimentation assaysof actin and Daam2-C at the indicated concentrations.Inverted still images from time-lapse TIRF movies using 0.3 µM F-actinin the presence of Daam2-C or fascin at 300 s. Scale bar, 10 µm.Low-speed sedimentation assays using 5 µM actin and 1 µM fascin. Forand, proteins in the pelletand supernatantfractions were recovered after centrifugation at 18,000 x g, resolved by SDS-PAGE and stained with Coomassie blue. Supplementary Fig. 8. Verification of the presence of Daam1-N and Daam1-C within the purified, autoinhibited Daam1 complex.The presence of His-tagged Daam1-N was confirmed by immunoblotting using a monoclonal anti-His antibody, andthe presence of Daam1-C was verified by immunoblotting using a polyclonal Daam1 antibody raised against the C-terminal half of Daam1. Supplementary Fig. 9. Constitutively-active Rif accumulates at the rear cortex of randomly migrating B16-F1 cells. Representative images of fixed B16-F1 cells on laminin transiently expressing EGFP-Rif-L77 and stained for F-actin with phalloidin. Scale bar, 10 µm. Supplementary Fig. 10. The activation of the autoinhibited Daam1 complex results in filament elongation rates comparable to those of the unregulated Daam1-C fragment. Actin elongation rates were quantified from TIRF movies using 1 µM actinand 3 µM PFN1, in the absence or presence of 1 nM Daam1-C or 1 nM Daam1 complex without or with 1 µM of respective constitutively-active GTPase, as indicated. At least eight filaments were analyzed from at least three independent movies in each condition. *P*-values were determined by one-way Anova. Data are shown as mean ± SD. *** *P*<0.001, ns: not significant. Supplementary Fig. 11. Active Rho-GTPases that interact with FMNL2-N1 release autoinhibition of the FMNL2 complex.GST pull-down experiments using constitutively-activeand dominant-negativeRho-GTPases as indicated confirm specific interaction with FMNL2-N1, with the exception of dominant-negative Cdc42, which also interacted with FMNL2-N1.Representative TIRF-microscopy images confirm activation of the autoinhibited FMNL2 complex by the respective Rho-GTPases at the single-filament level. Actin polymerization of 1 µM actinand 3 µM PFN1, in the absenceor presence of 1 nM FMNL2 complex withoutor with 1 µM of respective GTPase, as indicated. Data were monitored at 5 s intervals for 10 min and numbers in upper right corners of each panel indicate time in s; scale bar, 10 µm. Supplementary Fig. 12. FMNL2 interacts with constitutively-active variants of RhoA, Rac1 and Cdc42.Sedimentation of the purified components alone.Sedimentation velocity experiments to investigate the interactions of FMNL2-N1 with active Rho-GTPases, as indicated. Upon incubation of 9 µM FMNL2-N1 with 18 µM RhoA, 18 µM Rac1or 16 µM Cdc42, the *c*distribution of FMNL2-N1 shifted to higher s-values, demonstrating complex formation with all three GTPases. Note, heterogeneous sedimentation of FMNL2-N1 and the formed complexes.Upon incubation of 10 µM FMNL2-N2 with RhoA, Rac1, or Cdc42at the molar ratios as indicated, a concentration-dependent shift in the *c*distribution was observed.Integration of the *c*distributions shown in panels E-G was used to calculate *s*_w_ binding isotherms. Fitting of the isotherms with an A + B ⇌ AB model, yielded dissociation constants of ≤0.5 µM for Cdc42, 14 µM for RhoA and ≥150 µM for Rac1, respectively. Supplementary Figure 13. Confirmation of consecutive disruption of the *Daam1* and *Daam2* genes using the TIDE sequence trace decomposition tool. Panels display the corresponding frameshift mutations of *Daam1* allelesand *Daam2* alleles, indicated in base pairs, for the generated 1xKOand derived 2xKOcell lines, as indicated. R² denotes the goodness of fit. Supplementary Fig. 14. RNA-Seq analysis revealed that loss of Daam1/2 is not compensated by upregulation of other formins. RNA-Seq libraries were sequenced and analyzed as described. Note reduced abundance of Daam1 and Daam2 transcripts after genome editing, suggesting increased decay of the modified mRNAs. Data represent the arithmetic mean ± SD from three independent experiments. Supplementary Fig. 15. Daam1/2-deficient cells exhibit impaired FA disassembly despite unchanged assembly kinetics.FA assembly rates in control and mutant cells plated on laminin and transiently expressing EGFP-paxillin. Light blue and red lines denote logistic fits.Quantification of the FA assembly rates derived from data as shown in. Small dots represent measurements from individual FAs, while large dots represent the arithmetic mean of each biologically independent replicate. Lines and error bars indicate the mean ± SEM. A total of 30 FAs from five individual cells per cell line were analyzed. *N*, number of independent biological replicates. *P*-values were determined by two-tailed unpaired Student’s *t*-test based on the arithmetic means of the biological independent replicates. ns: not significant.FA disassembly rates in B16-F1 control and Daam1/2-KO cells transiently expressing EGFP-paxillin and migrating on laminin. Light blue and red lines depict exponential fits of the shown data. For quantification, see Fig. 7 O.Data points and error bars represent means ± SD from at least three biologically independent replicates each. Supplementary Fig. 16. Simultaneous disruption of the *Diaph1* and *Diaph2* genes, encoding mDia1 and mDia3, respectively, in Daam1/2-deficient B16-F1 cells, and of the *Daam1* and *Daam2* genes in FMNL2/3-deficient B16-F1 cells, as assessed by TIDE sequence trace decomposition and DNA sequencing. The corresponding frameshift mutations, indicated in base pairs, are shown for two independent clones of the generated 4xKO cell lines. R² denotes the goodness of fit. In quadruple mutant #8-33-26 lacking mDia1/3 and Daam1/2 expression, a fragment of approximately 400 bp was inserted into the *Diaph2* target site in all alleles, precluding TIDE-based analysis. Nevertheless, subsequent DNA sequencing revealed multiple stop codons leading to premature termination of *Diaph2* translation. Supplementary Fig. 17. Loss of mDia/Daam-family formins increases tension within the SF-FA system.Representative images of fixed control and mutant cells stained for the FA marker zyxin beforeand after binarization. Dashed orange lines indicate cell outlines. Scale bar, 10 μm.Quantification of zyxin intensity.Quantification of total FA area normalized to cell area.* P*-values in B and C were determined using Kruskal-Wallis ANOVA followed by Dunn’s multiple comparison test. Boxes in box plots indicate 50%and whiskers 90%of all measurements, with dashed red lines depicting the means. Medians are highlighted by indentation of boxes. *n*, number of cells analyzed. ** P<0.01, *** *P*<0.001, ns: not significant.

Supplementary Material 2. Movie 1. Daam1-C promotes nucleation of actin filaments in a concentration-dependent manner. Polymerization of 1 µM actinin the presence of 1, 2 or 5 nM Daam1-C. TIRF-images were captured at 5 s intervals for a period of 10 min. Time is indicated in min:s. Scale bar, 10 µm. Movie 2. Daam2-C promotes nucleation of actin filaments in a concentration-dependent manner. Polymerization of 1 µM actinin the presence of 1, 2 or 5 nM Daam2-C. TIRF-images were captured at 5 s intervals for a period of 10 min. Time is indicated in min:s. Scale bar, 10 µm. Movie 3. The strong nucleation activity of Daam1-C masks its bundling activity under normal conditions. Polymerization of 1 µM actinin the presence of 250 nM Daam1-C. TIRF-images were captured at 5 s intervals over a period of 10 min. Time is indicated in min:s. Scale bar, 10 µm. Movie 4. The C-terminal fragments of Daam1 and Daam2 effectively elongate actin filaments in the presence of PFN1. Polymerization of 1 µM actinin the presence of 3 µM PFN1 and 1 nM Daam1-C or Daam2-C, respectively. TIRF-images were captured at 5 s intervals for a period of 10 min. Time is indicated in min:s. Scale bar, 10 µm. Movie S5. Distinct Rho-GTPases are capable of releasing autoinhibition of the inherently inactive Daam1 complex. Polymerization of 1 µM actin, 3 µM PFN1 and 1nM Daam1 complex either in absence or presence of the active Rho-GTPases, as indicated. Note that addition of the GTPases activated the complex, as evidenced by the appearance of dim, fast-growing filaments characteristic of formin-mediated elongation. TIRF-images were captured at 5 s intervals for a period of 10 min. Time is indicated in min:s. Scale bar, 10 µm. Movie S6. Multiple Rho-GTPases are capable of releasing autoinhibition of the inactive FMNL2 sandwich complex. Polymerization of 1 µM actin, 3 µM PFN1 and the inactive FMNL2 complex either in absence or presence of indicated Rho-GTPases. TIRF-images were captured at 5 s intervals for a period of 10 min. Time is indicated in min:s. Scale bar, 10 µm. Movie 7. Daam1/2-deficient B16-F1 cells form normal microspikes. B16-F1 and Daam1/2-KO cells were transfected with EGFP-fascin to monitor microspike dynamics during migration on laminin using time-lapse microscopy. Time is indicated in min:s. Scale bar, 10 µm. Movie 8. Combined loss of Daam1 and Daam2 does not affect lamellipodial actin polymerization. B16-F1 and mutant cells transiently expressing EGFP-β-actin were used to analyze lamellipodial actin polymerization during random migration on laminin using FRAP. The photobleached area is indicated by a white rectangle. Time is indicated in s. Scale bar, 5 µm. Movie S9. Loss of Daam1 and Daam2 alters cell morphology and the ability to retract. B16-F1 and Daam1/2-KO cells were recorded using time-lapse phase-contrast imaging while randomly migrating on laminin. Note the difference between control and mutant cells with respect to their morphology and capability to retract the cell rear. Time is indicated in min:s. Scale bar, 50 µm. Movie 10. Combined loss of Daam1 and Daam2 in B16-F1 cells results in diminished focal-adhesion turnover, disassembly rates, and sliding velocities. Representative TIRF movies show B16-F1 and Daam1/2-KO cells transiently expressing EGFP-paxillin during random migration on laminin. Time is indicated in min:s. Scale bar, 10 µm. Movie 11. Consecutive loss of cortical formins results in extensive blebbing. Combined loss of Daam formins with either FMNL2/3 or mDia1/3 caused frequent and extensive blebbing, with mDia/Daam 4xKO cells exhibiting the most severe cortical defects. Representative DIC time-lapse images depict control and mutant cells on glass. Time is indicated in min:s. Scale bar, 20 µm. Movie 12. Ectopic expression of EGFP-tagged full-length formins rescues the blebbing phenotype. FMNL/Daam 4xKO and mDia/Daam 4xKO cells were transfected with EGFP-tagged full-length formin constructs as indicated, seeded on glass, and analyzed by DIC time-lapse microscopy. Corresponding fluorescence still images shown on the left, acquired before movie recording, confirm expression of the respective EGFP-formin construct in the cells. The corresponding DIC movies are shown on the right. Time is indicated in minutes. Scale bar, 20 µm. Movie 13. Loss of cortical formins leads to prolonged duration of mitotic cell division. Phase-contrast imaging of dividing control and formin-KO cells on laminin. Note that all cell lines round up upon entry into mitosis at ~60 min, but differ in the time required to complete daughter cell separation. Time is indicated in h:min:s. Scale bar, 20 μm. Movie 14. NM2A prominently localizes to the rear cortex and SFs in FMNL/Daam and mDia/Daam 4xKO mutant cells during rear-end retraction. B16-F1 wild type, FMNL/Daam 4xKO, and mDia/Daam 4xKO cells transiently expressing EGFP-NM2Aand mCherry-LifeActwere imaged during rear retraction while randomly migrating on laminin. Brightest-point projection of confocal stacks are shown. Time is indicated in min:s. Scale bar, 15 µm. Movie 15. Combined loss of mDia1/3 and Daam1/2 results in extensive rear blebbing in migrating cells. B16-F1 wild-type, mDia1/3-KO, FMNL/Daam 4xKO and mDia/Daam 4xKO cells ectopically expressing EGFP-NM2A were imaged during random 2D-migration on laminin. In stark contrast to the other cell lines, only the mDia/Daam 4xKO mutant cells exhibited continuous rear blebbing, consistent with severe cortical impairment that renders the rear cortex unable to withstand NM2-generated forces and the resulting hydrostatic pressure. Movies depict single confocal sections recorded approximately 2 µm above the substratum. Time is indicated in min:s. Scale bar, 10 µm

## Data Availability

All data needed to evaluate the conclusions in the paper are present in the paper and/or the Supplementary Materials.
