# Supplementary material for: Daam-, FMNL-, and mDia-family formins jointly regulate cortical actin dynamics in melanoma cells
Source: Cell Commun Signal. 2026 Oct 1;24:527. doi: 10.1186/s12964-026-03229-9 (PMC13632457; doi:10.1186/s12964-026-03229-9)

Supplementary information:

Fig. 2C

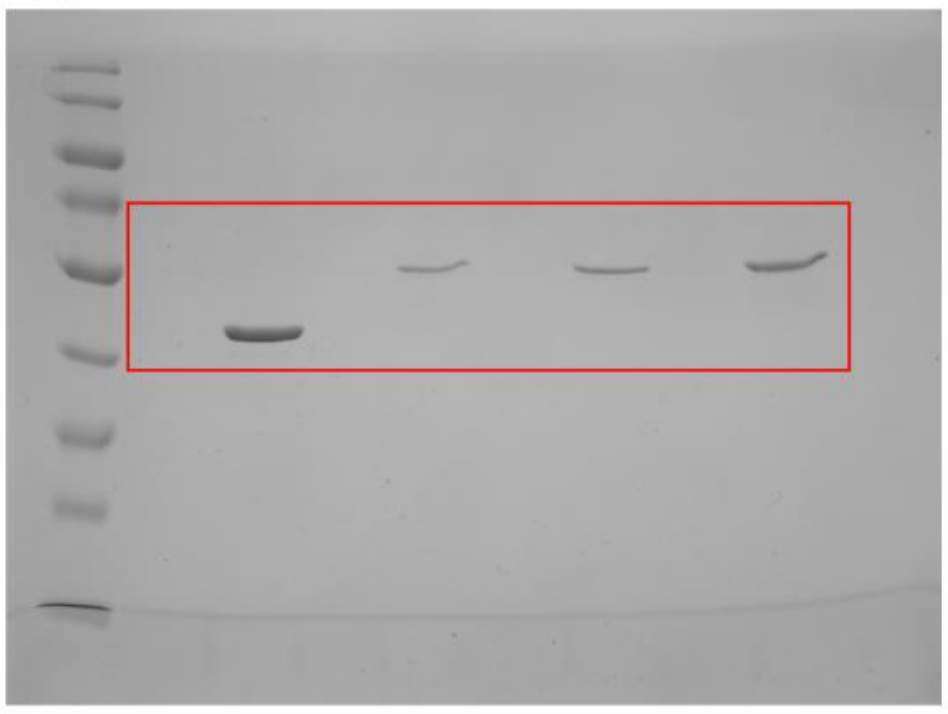

Fig. 2C

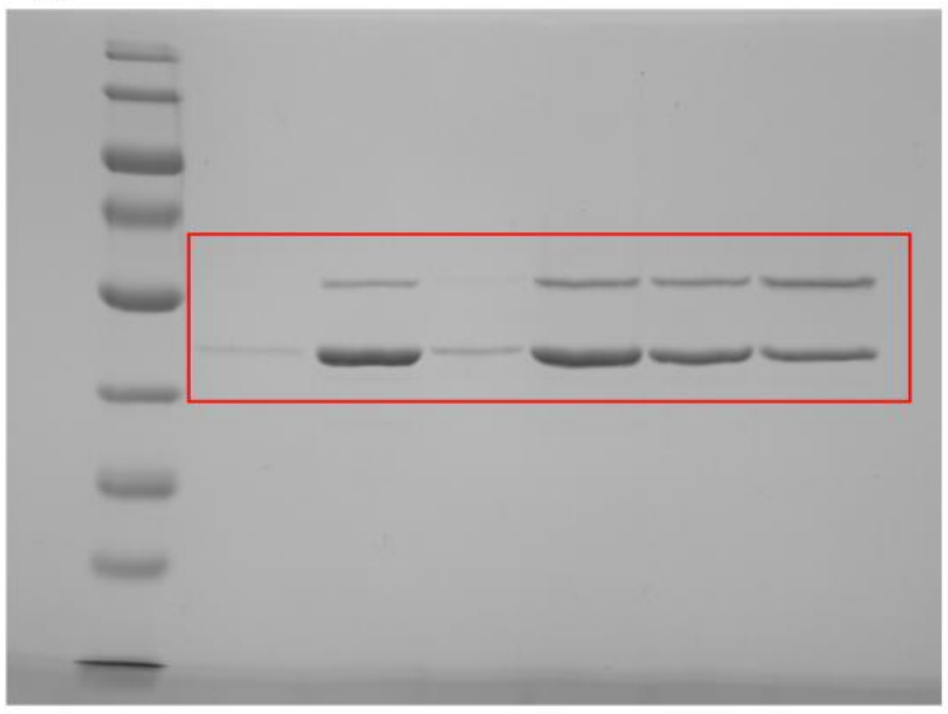

Fig. 2E

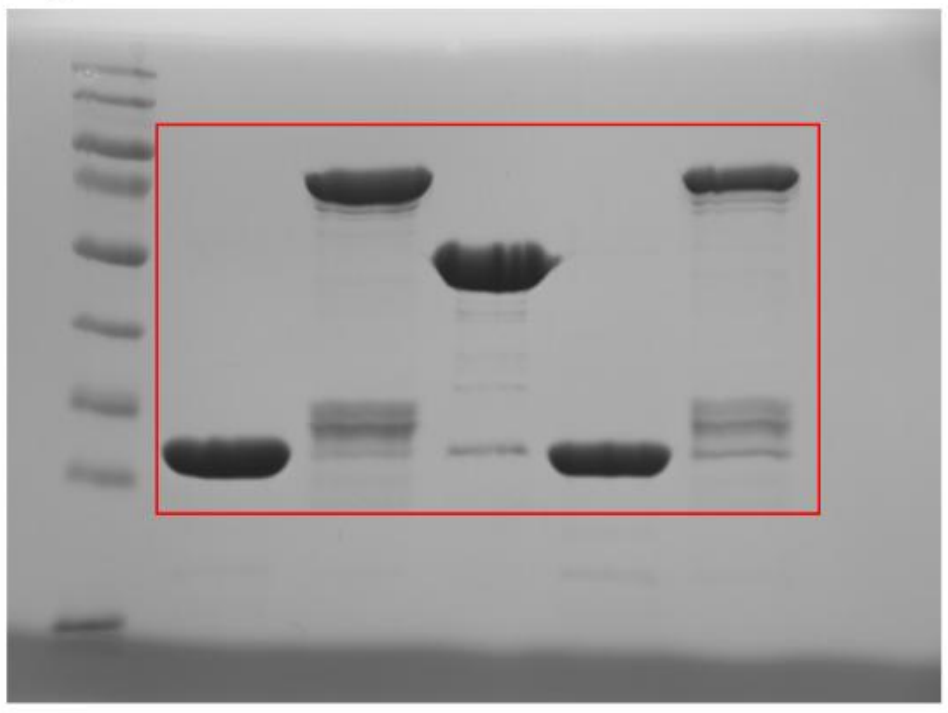

Fig. 3A

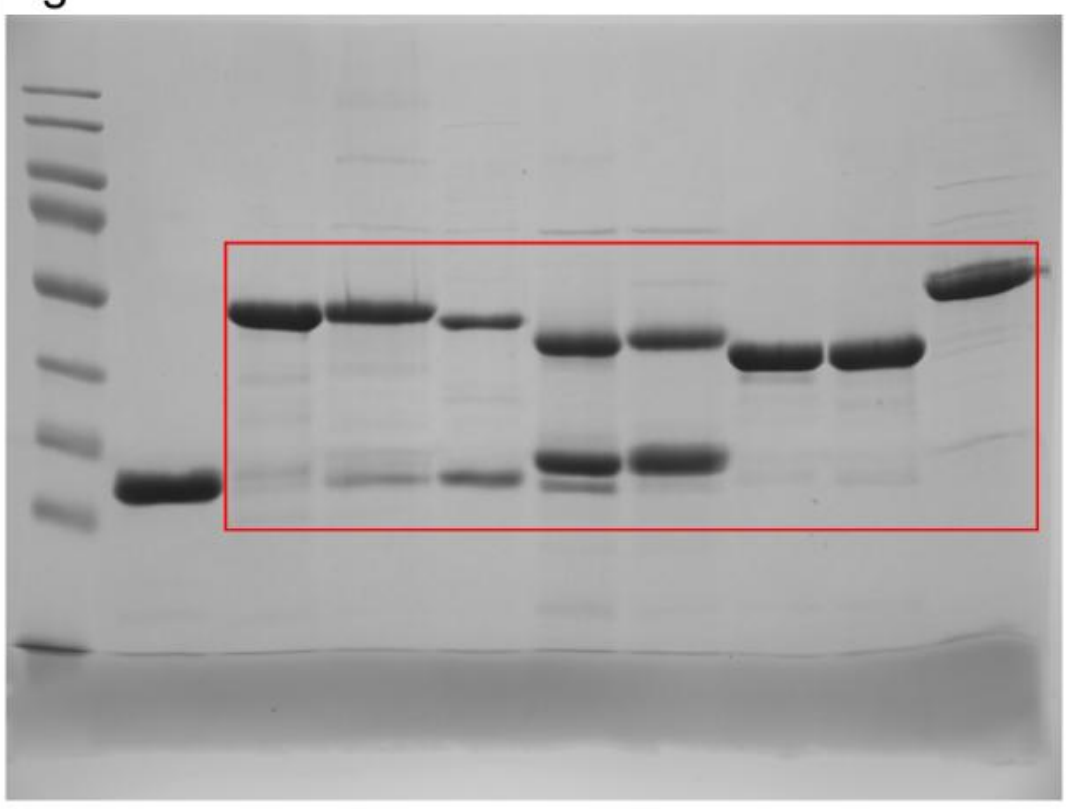

Fig. 3B

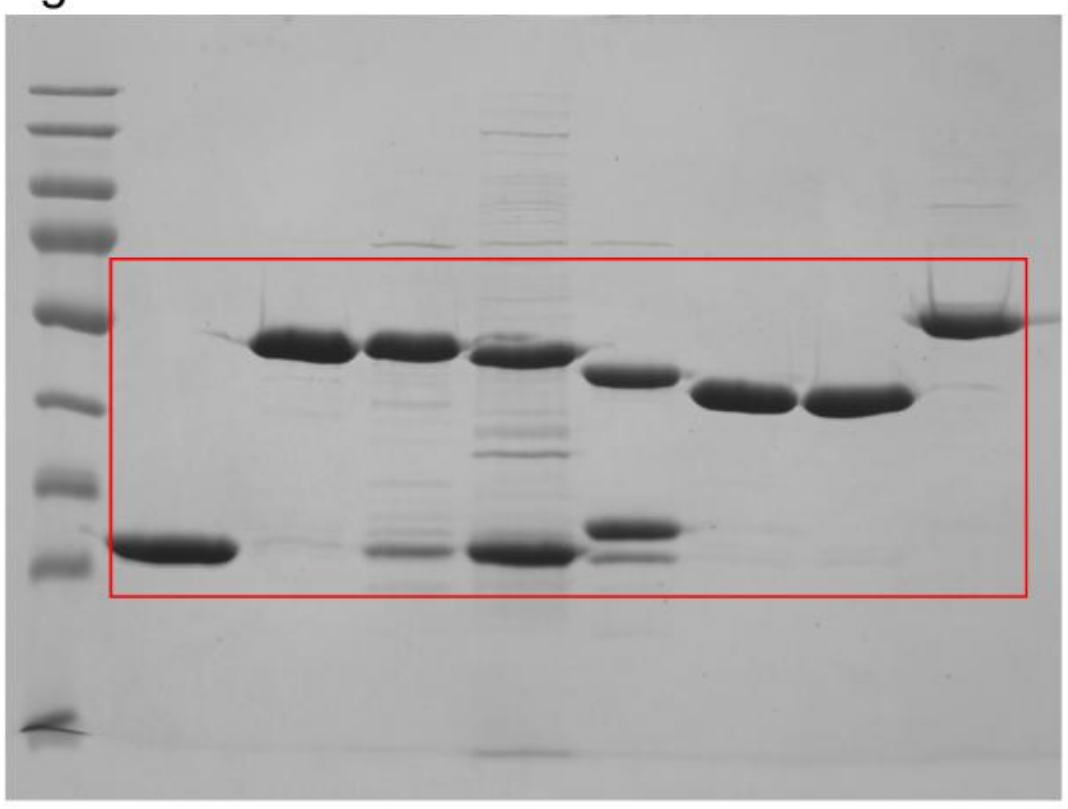

Fig. 3C

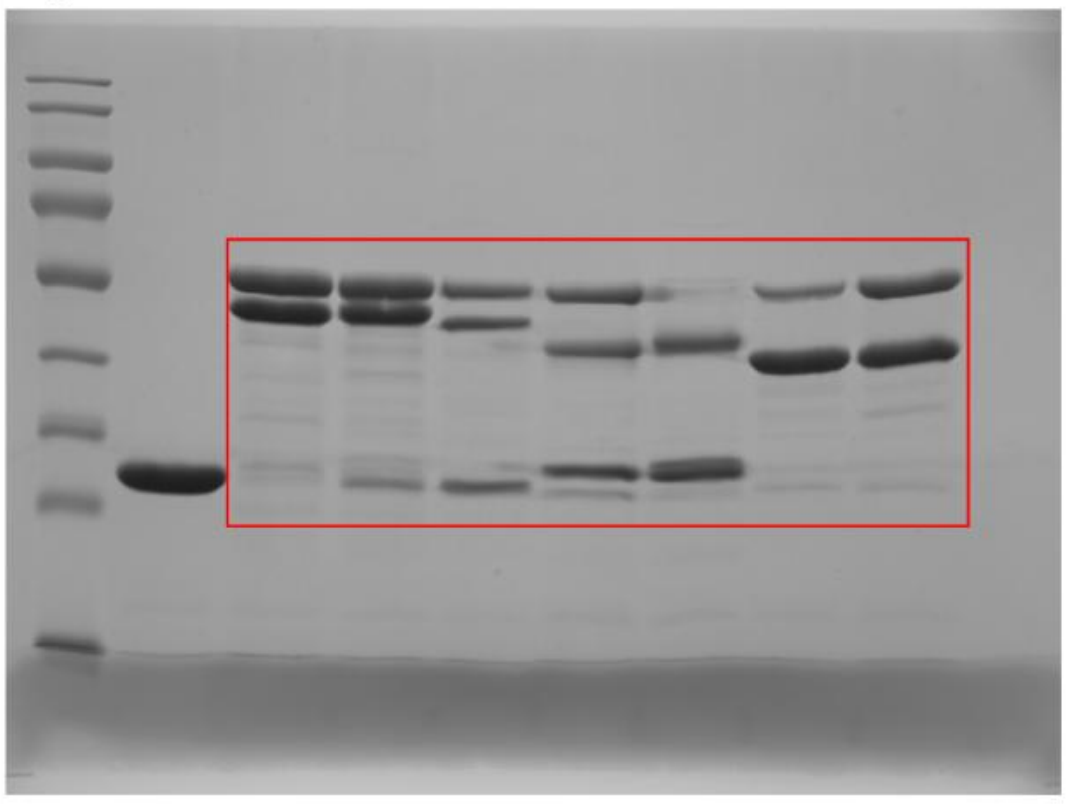

Fig. 3D

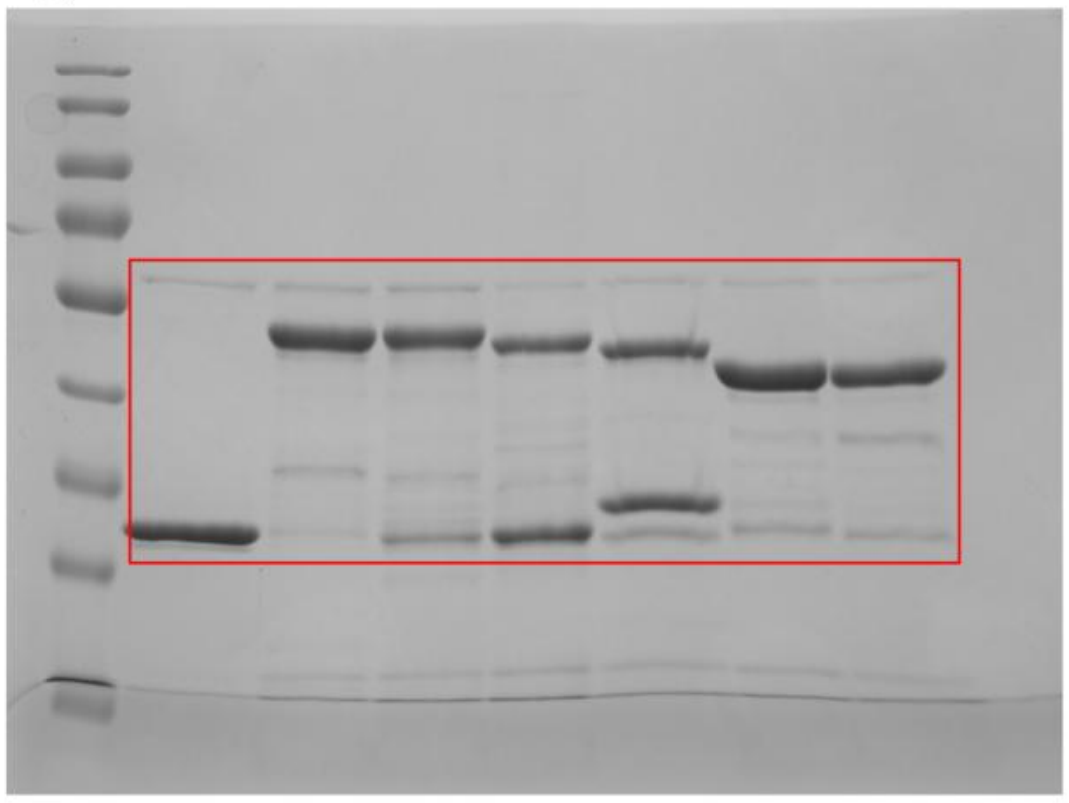

Fig. 4A

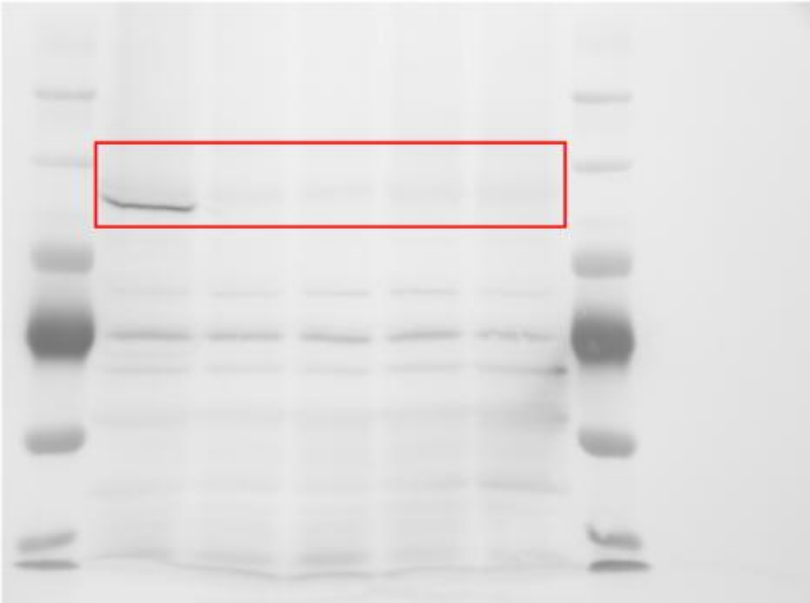

Fig. 4A

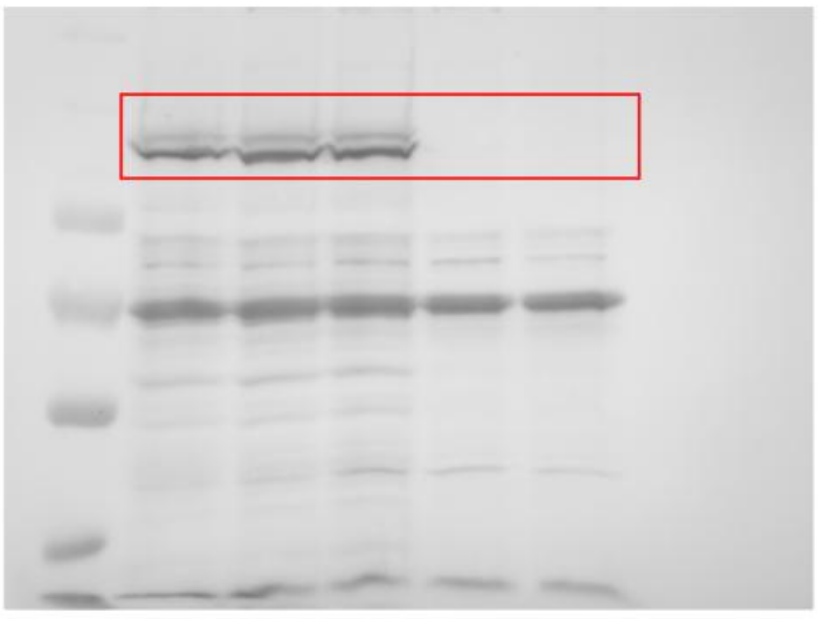

Fig. 4A

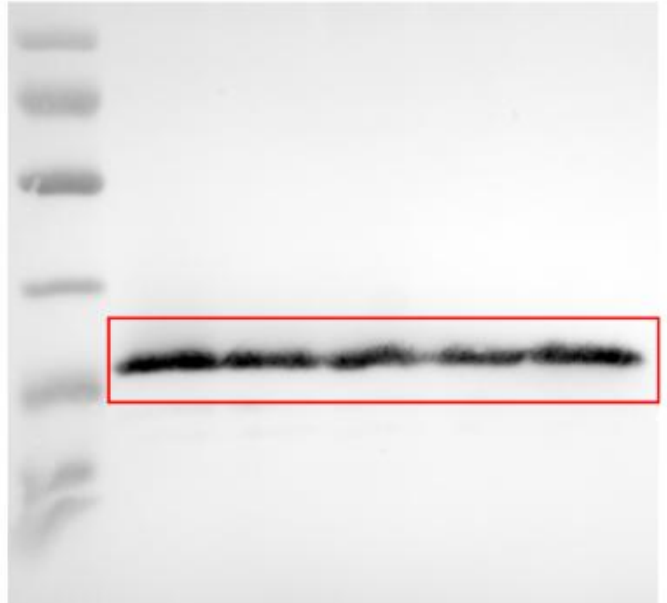

Fig. 7A

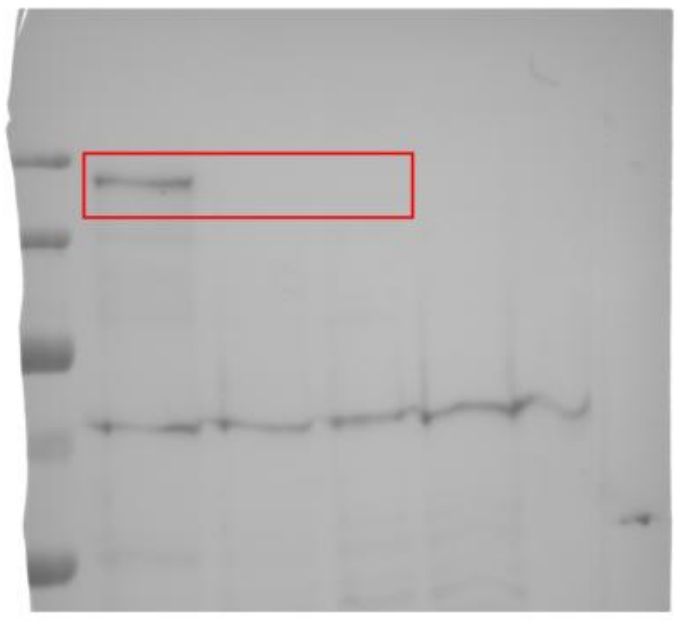

Fig. 7A

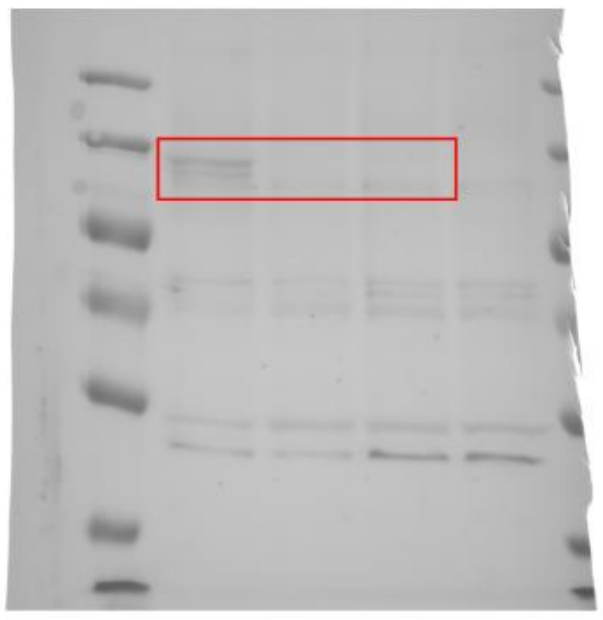

Fig. 7A

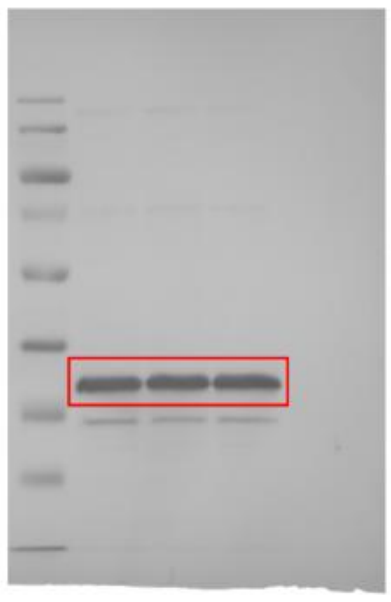

Fig. 7B

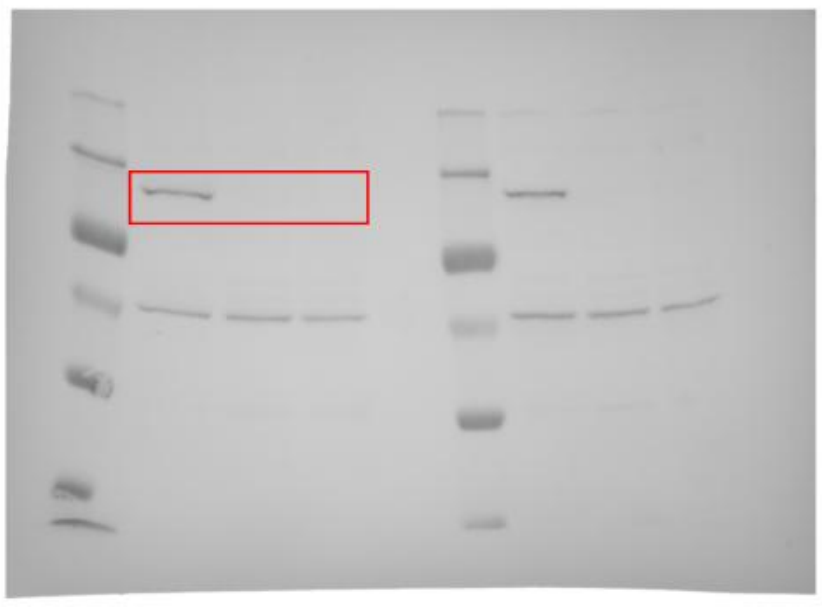

Fig. 7B

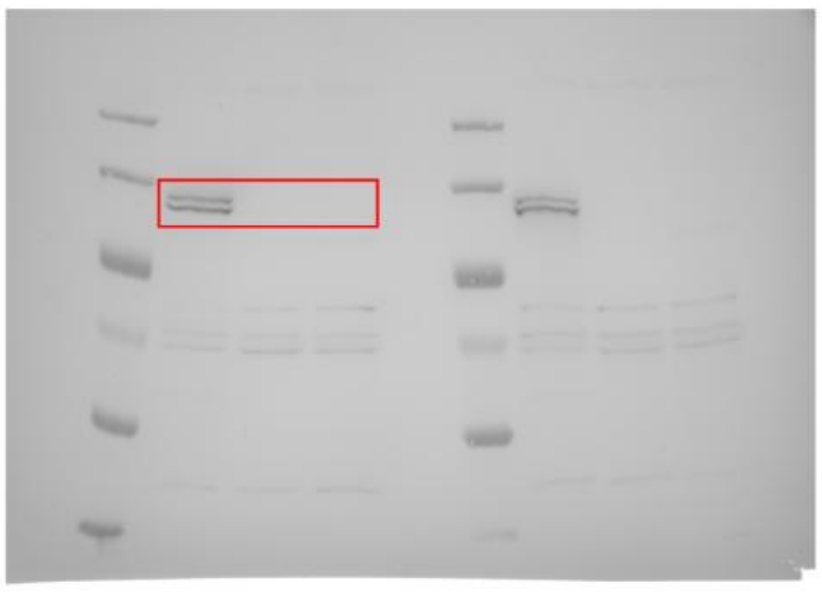

Fig. 7B

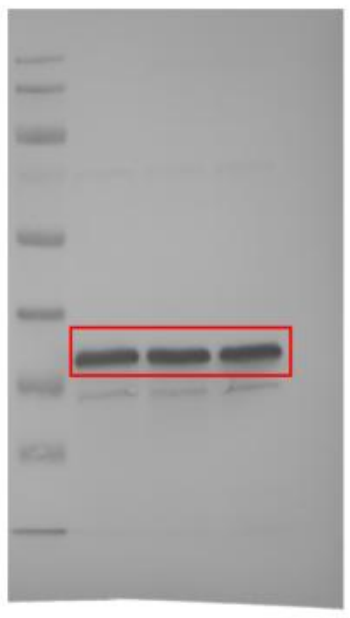

Fig. 8E

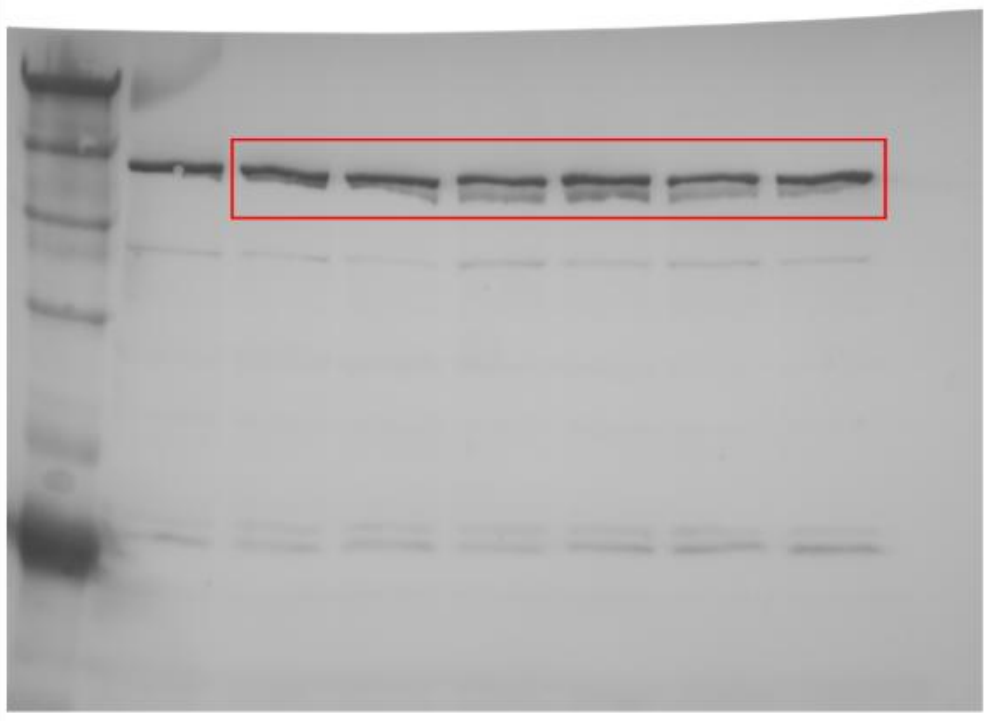

Fig. 8E

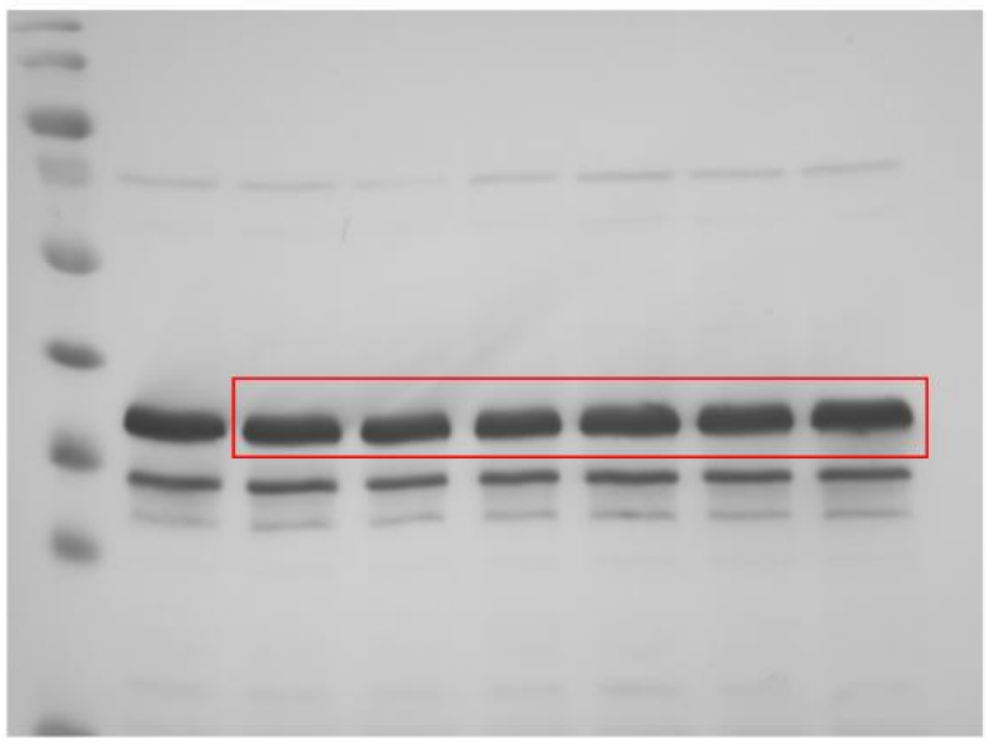

Supplementary Fig. 2A

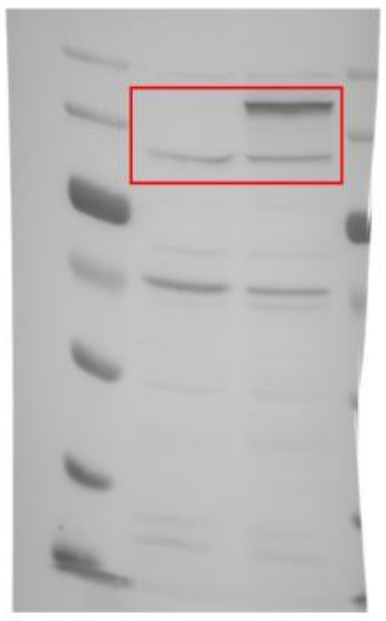

Supplementary Fig. 2A

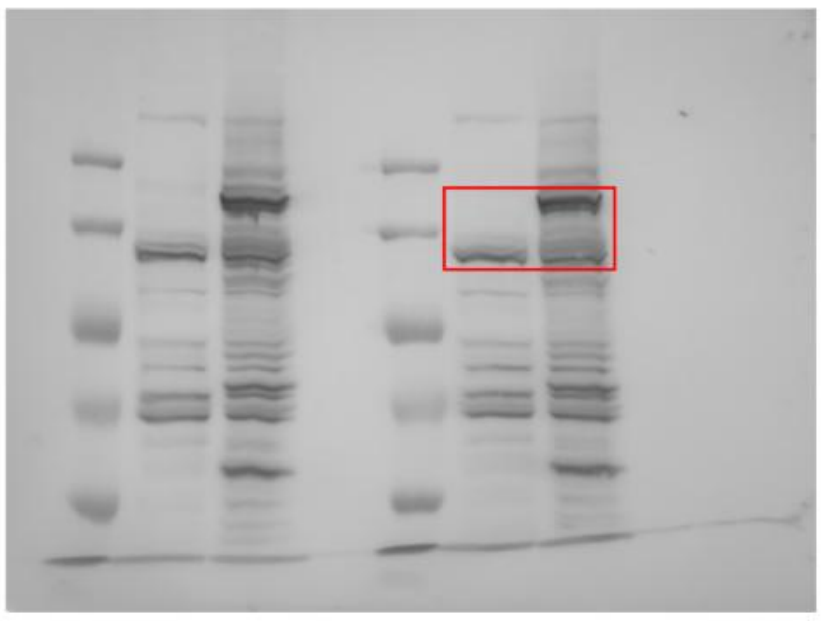

Supplementary Fig. 2A

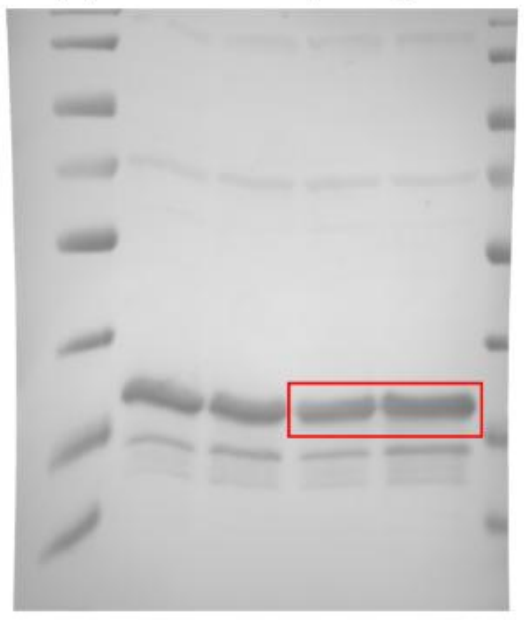

Supplementary Fig. 2A

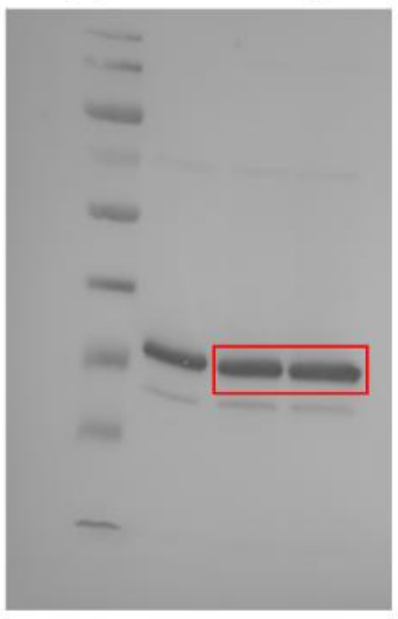

Supplementary Fig. 7A

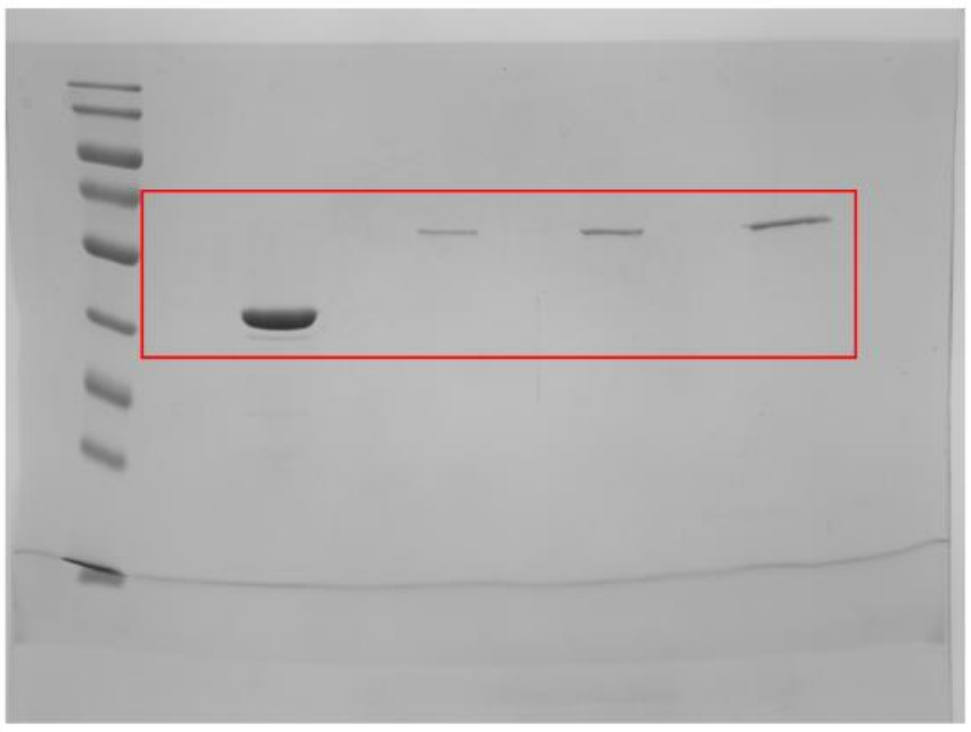

Supplementary Fig. 7A

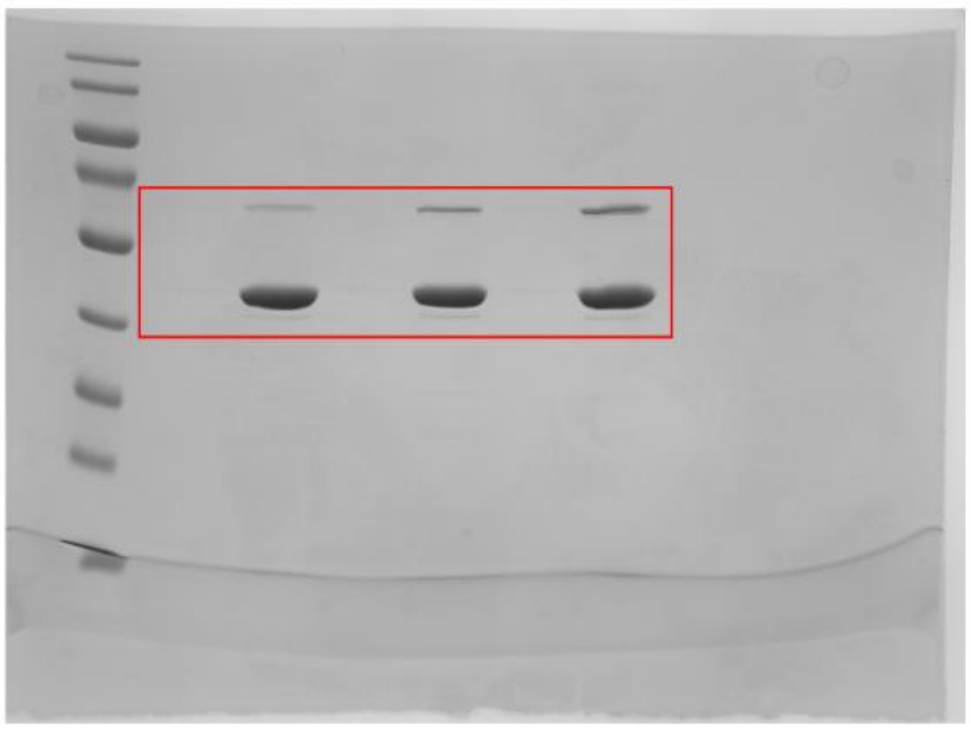

Supplementary Fig. 7C

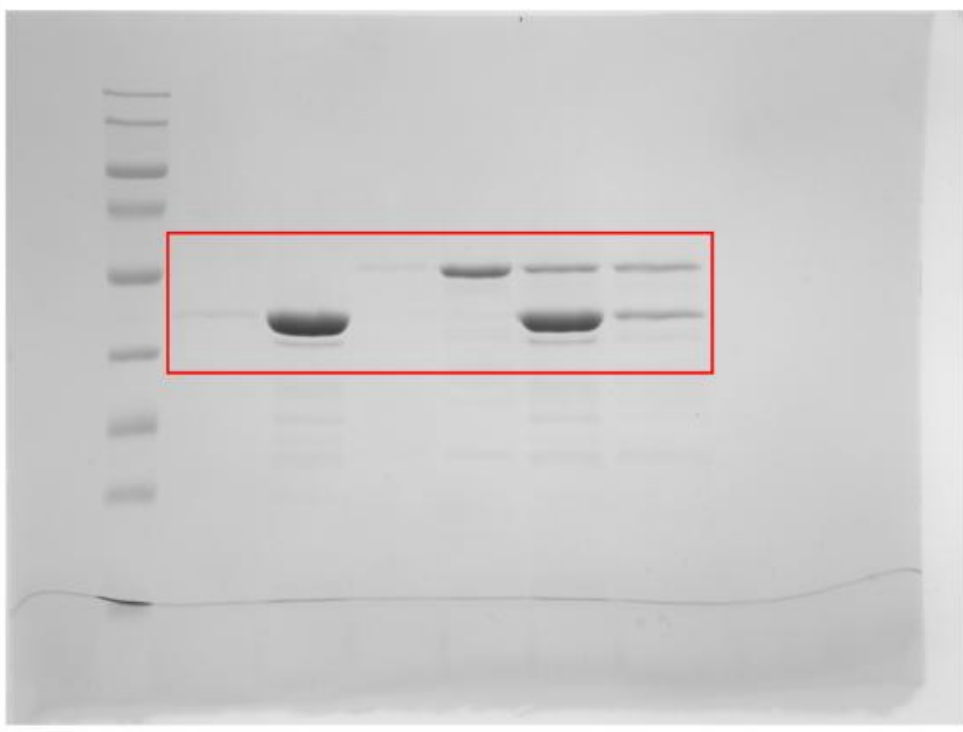

Supplementary Fig. 8A

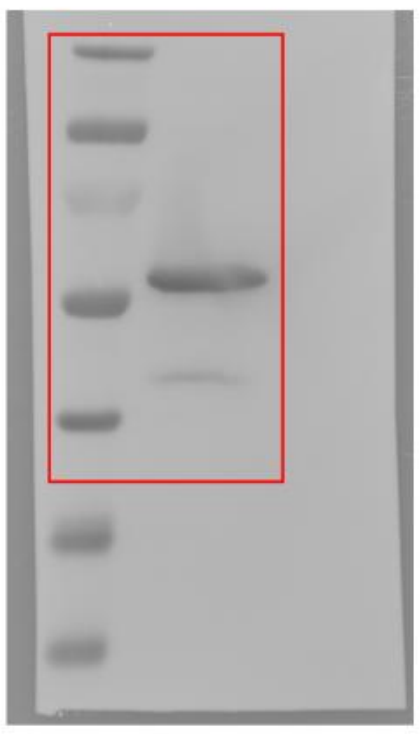

Supplementary Fig. 8B

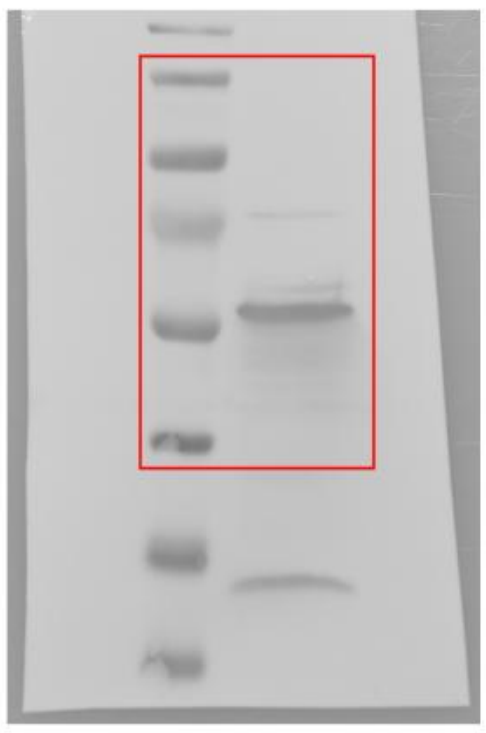

Supplementary Fig. 11A

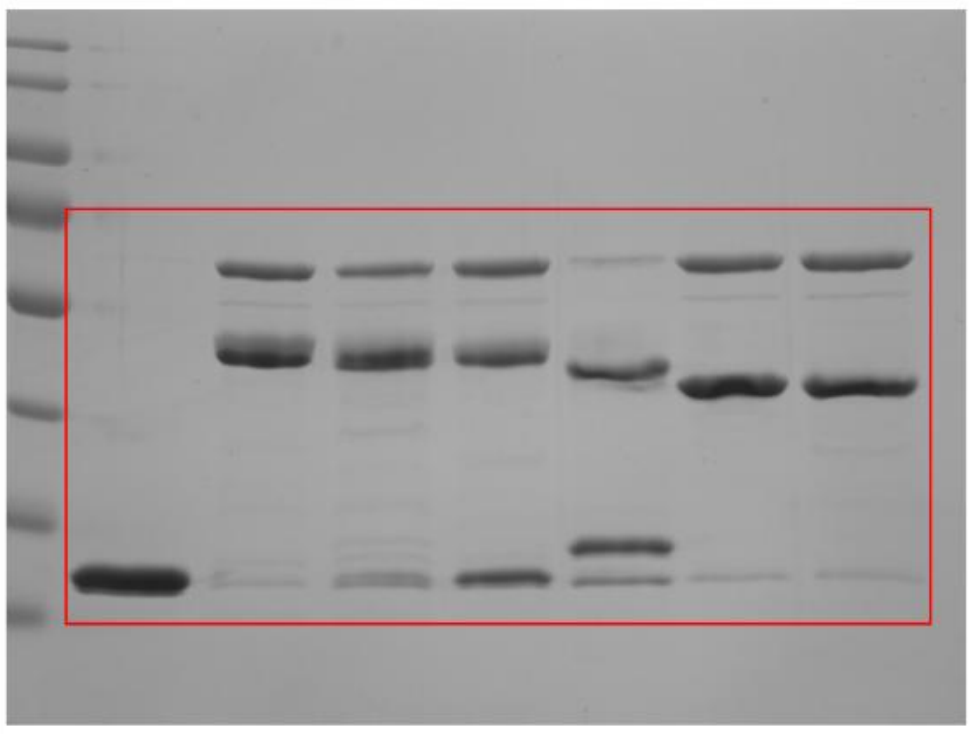

Supplementary Fig. 11B

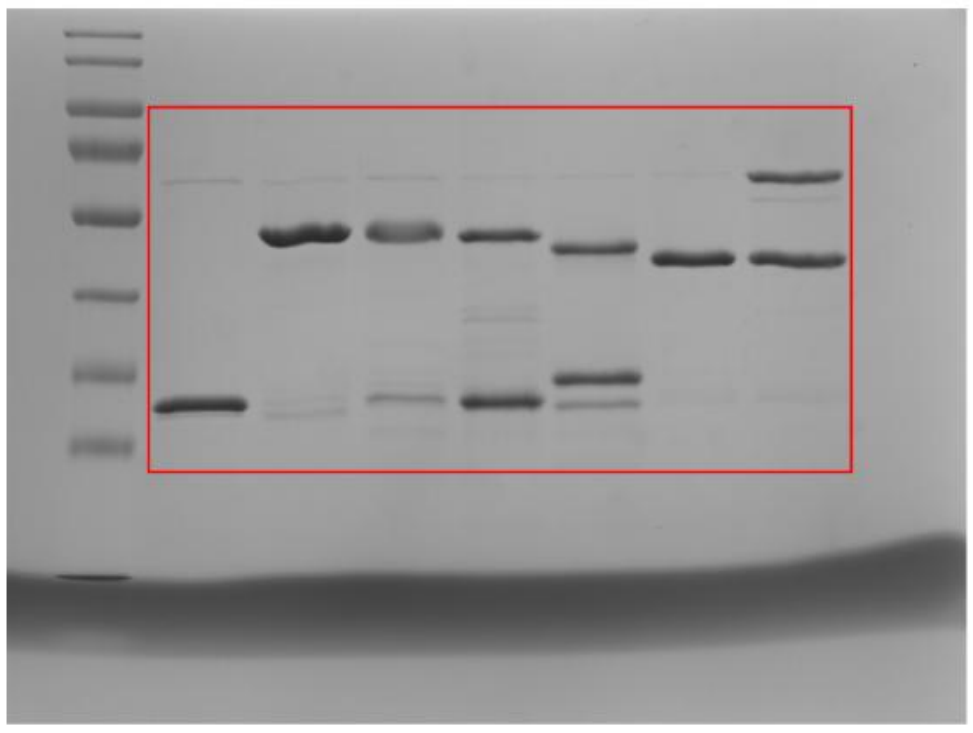

Supplement: Supplementary file 1 [file 12964_2026_3229_MOESM1_ESM.pdf]
